# Mammalian RAD51 paralogs protect nascent DNA at stalled forks and mediate replication restart

**DOI:** 10.1093/nar/gkv880

**Published:** 2015-09-09

**Authors:** Kumar Somyajit, Sneha Saxena, Sharath Babu, Anup Mishra, Ganesh Nagaraju

**Affiliations:** Department of Biochemistry, Indian Institute of Science, Bangalore-560012, India

## Abstract

Mammalian RAD51 paralogs are implicated in the repair of collapsed replication forks by homologous recombination. However, their physiological roles in replication fork maintenance prior to fork collapse remain obscure. Here, we report on the role of RAD51 paralogs in short-term replicative stress devoid of DSBs. We show that RAD51 paralogs localize to nascent DNA and common fragile sites upon replication fork stalling. Strikingly, RAD51 paralogs deficient cells exhibit elevated levels of 53BP1 nuclear bodies and increased DSB formation, the latter being attributed to extensive degradation of nascent DNA at stalled forks. RAD51C and XRCC3 promote the restart of stalled replication in an ATP hydrolysis dependent manner by disengaging RAD51 and other RAD51 paralogs from the halted forks. Notably, we find that Fanconi anemia (FA)-like disorder and breast and ovarian cancer patient derived mutations of RAD51C fails to protect replication fork, exhibit under-replicated genomic regions and elevated micro-nucleation. Taken together, RAD51 paralogs prevent degradation of stalled forks and promote the restart of halted replication to avoid replication fork collapse, thereby maintaining genomic integrity and suppressing tumorigenesis.

## INTRODUCTION

The phenomenon of chromosomal instability (CIN) is a hallmark of nearly all cancer types (1–4). CIN develops at early stages of cancer, and replication stress in the form of fork stalling is proposed to be the prominent driving force for this instability (5–8). The link between replication stalling to tumor development is more appreciated after the observation that oncogene activation induces replication stress (9,10), specifically by the depletion of nucleotide pool in precancerous cells (11,12). The RAD51 recombinase, a key player in recombinational repair of DNA double-strand breaks (DSBs) participates in the replication fork maintenance (13). In addition, recent studies have clearly established the role of Fanconi anemia (FA)-BRCA tumor suppressors in preventing genomic instability upon replication stalling caused by various endogenous and exogenous replication poisons (14–19). However, maintenance of stalled replication forks, and the regulation of continuous DNA synthesis from the halted replication demands more mechanistic studies and the associated factors related to FA-BRCA-RAD51 proteins.

Mammalian genome encodes for five RAD51 paralogs; RAD51B, RAD51C, RAD51D, XRCC2 and XRCC3 (20–22). These paralogs have been implicated in homologous recombination (HR) mediated repair of DSBs and DNA damage signaling (21,23–26). Mouse knockout of RAD51 paralogs causes early embryonic lethality (20,21,27–30). Despite their identification over nearly two decades ago, their precise roles in genome maintenance are less understood. Interestingly, mono-allelic germline mutations in all five *RAD51* paralogs are known to cause various types of cancer including breast and ovarian cancer (31–36). In addition, FA-like disorder with bi-allelic germline mutations in *RAD51C*, and *XRCC2* also has been reported (37,38). The tumor suppressor functions of RAD51 paralogs have been attributed to their role in DSB repair by HR and DNA damage signaling (20,21,23–26,39,40). Recent studies show that FA core complex proteins, FANCD2 and BRCA2 protect forks after HU induced fork stalling in an HR independent manner, but are dispensable for promoting replication restart (14–16,19). Interestingly, there are handful of interesting reports which suggest the involvement of RAD51 paralogs in the maintenance of replication forks in challenged or unchallenged conditions (25,26,39,41–46). Specifically, RAD51 and XRCC3 have been shown to restrain fork progression upon DNA damage by cisplatin or UV (43), and promote replication restart after pulse treatment with HU (15,44,47–49). However, the link between the mechanism of fork stability and its restart during perturbed replication, and the systematic role(s) of RAD51 paralogs in connecting these events remains enigmatic.

In this study, we report a previously unanticipated role of RAD51 paralogs in preventing DSB generation at the stalled forks and mediating continuous DNA synthesis. RAD51 paralogs in pre-assembled distinct complexes localize to the stalled replication forks through their direct interaction with nascent strands. In parallel to FA-BRCA proteins, binding of RAD51 paralogs at the nascent DNA protects the stalled forks from the action of MRE11, and keeps them viable for replication resumption. We find that RAD51C and XRCC3, but not XRCC2 mediated ATP hydrolysis drives continuous DNA synthesis from the stalled site by disengaging nascent strand bound RAD51 and RAD51 paralogs upon replication recovery. This function of RAD51 paralogs is distinct from those of FA-BRCA proteins in the fork maintenance, and brings about the mechanistic link between the outcomes of stable stalled replication forks toward its restart. Finally, our data with patient derived mutants of RAD51C uncover the tumor suppressor function (s) of RAD51 paralogs, at least in part mediated by suppression of replication associated damage and promotion of timely restart to avoid error prone repair mechanisms.

## MATERIALS AND METHODS

### Cell lines, cell culture and transfections

Human cell lines HeLa and U2OS, the Chinese hamster cell lines CL-V4B (RAD51C^−/−^), irs1 (XRCC2^−/−^), irs1-SF (XRCC3^−/−^) and their respective parental cells V79B, V79 and CHO-AA8, respectively and BRCA2 deficient Chinese hamster cells V-C8 cells were grown in Dulbecco's modified Eagle's medium supplemented with 10% fetal bovine serum at 37°C in a humidified air containing 5% CO_2_. All plasmid transfections for stable and transient expression were performed using a Bio-Rad gene pulsar X cell (250 V and 950 μF).

### DNA constructs and statistical tests

Human RAD51 paralogs RAD51C, XRCC2 and XRCC3 WT and mutant constructs were generated using polymerase chain reaction (PCR)-based mutagenesis and cloned into the pcDNA3β vector (25,26). hXRCC3, hRAD51C, hXRCC2, and FANCM shRNA constructs were generated using reported sequences (25,50) and cloned into the pRS shRNA vector. *P*-values are obtained by performing student t-tests.

### Cell synchronization and cell cycle analysis

Cells were synchronized at various phases such as G0/G1-phase by addition of serum starved media for 48 h, S-phase by thymidine block (2 mM) for 14 h followed by two washes in PBS and cells were released into fresh medium for 11 h, and then arrested a second time with 1 μg/ml aphidicolin for 12 h, M-phase by nocodazole (150 ng/ml) for 14 h and released in fresh media for 1 h. Alternatively, cells were synchronized at the G2/M phase by the addition of nocodazole. The floating mitotic cells were then collected, washed with fresh media and then replated. The cells were collected after 1 h (predominantly M phase), 4 h (predominantly G1 phase) and 12 h (predominantly S/G2 phase) and processed for analyzing the cell cycle stages. Collected single-cell suspensions were fixed overnight with 70% ethanol in PBS at −20°C. After centrifugation, the cells were incubated with 0.10 mg/ml RNaseA (Fermentas) in PBS at 42°C for 4 h and then incubated for 10 min with 50 μg/ml propidium iodide (PI) in dark. A total of 1×10^4^ cells were analyzed by Canto flow cytometer (BD Biosciences). Aggregates were gated out and percentage of cells with 2N and 4N DNA content was calculated using FACSDiva Version 6.1.1 software (BD Biosciences).

### Immunoprecipitation, western blotting and antibodies

Cells were harvested and lysed in RIPA buffer (without SDS) supplemented with a complete protease and phosphatase inhibitor cocktail (Roche). For the immunoprecipitation assays, cell lysates were incubated with indicated antibodies using protein A/G beads. The proteins were resolved on a 10% SDS-PAGE gel and transferred onto PVDF membrane (Millipore). The membranes were blocked using 3% BSA in TBST (50 mM Tris-HCl pH 8.0, 150 mM NaCl, 0.1% tween-20) and incubated with primary antibody for 12 h at 4^0^ C. The primary antibodies against RAD51 (1:500), RAD51B (1:250), RAD51C (1:500), RAD51D (1:100), XRCC2 (1:250), XRCC3 (1:200), ORC2 (1:500), H2AX (1:250), P-Histone (S10) (1:1000), FANCD2 (1:100), FANCM (1:50), RPA1 (1:250), RPA2 (1:250), Histone-H3 (1:250), MCM2 (1:250), MCM3 (1:250), MCM10 (1:250), CDC45 (1:250), MUS81 (1:250), SLX4 (1:250), FANCJ (1:250), BLM (1:100), WRN (1:250), Lamin A (1:200), CHK1P (S345) (1:200), CHK2P (T68) (1:150), H2A (1:250), β-actin (1:2000) and α-tubulin (1:2500) that were used for western blot analysis were purchased from Santa Cruz. The anti-MRE11 (1:200) and anti-γ-H2AX (1:1000) antibodies were obtained from BD Biosciences, and the anti-PCNA (1:2000) antibody was obtained from cell signaling technology. The membranes were incubated with HRP-conjugated secondary antibodies, developed by chemiluminescence and imaged using Chemidoc (GE healthcare LAS 4000).

### Chromatin fractionation and IdU Co-immunoprecipitation of proteins present at the replication forks

After HU treatment, U2OS or HeLa cells (10 × 10^6^) were labeled with 100 μM IdU for 40 min. Cells were then cross-linked in 1% paraformaldehyde for 20 min. The cytosolic protein fraction was removed by incubation in hypotonic buffer (10 mM HEPES, pH 7, 50 mM NaCl, 0.3 M sucrose, 0.5% Triton X-100, supplemented with protease inhibitor; Roche) for 15 min on ice and centrifuged at 1500xg for 5 min. The soluble nuclear fraction was removed by incubation with nuclear buffer (10 mM HEPES, pH 7, 200 mM NaCl, 1 mM EDTA, 0.5% NP-40 and protease inhibitor cocktail) for 10 min on ice and then centrifuged at 13 000 rpm for 2 min. The pellets were resuspended in lysis buffer (10 mM HEPES, pH 7, 500 mM NaCl, 1 mM EDTA, 1% NP-40 and protease inhibitor cocktail), sonicated at low amplitude and centrifuged for 1 min at 13 000 rpm; the supernatant was then transferred to a new tube. Total chromatin protein was quantified using the standard Bradford's method, and a total of 250 μg protein was used for IP with 5 μg anti-IdU antibody and 20 μl of Protein G- agarose (GE heathcare). The IP reaction was washed twice with nuclear buffer and twice with washing buffer (10 mM HEPES and 0.1 mM EDTA protease inhibitor cocktail), incubated in 2× sample loading buffer (100 mM Tris HCl [pH 6.8], 100 mM DTT, 4% SDS, 0.2% bromophenol blue, and 20% glycerol) for 30 min at 90°C, and was used for Western Blot.

### Immunofluorescence

Exponentially growing cells were seeded onto coverslips, then treated (or mock-treated) with the indicated DNA damaging agent. After treatment, the cells were washed with PBS and fixed in 3.7% formaldehyde 10 min at room temperature followed by 90% methanol for 5 min. Later cells were blocked in 0.5% BSA/0.5% TritonX-100 for 30 min. The cells were then incubated with the indicated primary antibodies and FITC/TRITC-conjugated secondary antibodies (Sigma) for 1 h each at room temperature, and then stained with PI/DAPI before mounting onto slides. Cells were acquired using Carl Zeiss confocal microscope and images were processed using Zeiss LSM image browser software.

DNA replication sites were visualized by incorporation of chlorodeoxyuridine (CldU) and iododeoxyuridine (IdU) into DNA. Indicated cells were grown onto the coverslips and labeled with 100 μM CldU (Sigma) or IdU (Sigma) for 30 min at different time intervals. Cells were washed with PBS, fixed with 70% cold ethanol, and stored at 4°C.For antibody staining, the ethanol was removed, and 90% methanol was added for 5 min. Cells were washed twice with PBS and incubated with 1.5 N HCl for 40 min to denature the DNA. Cells were washed with PBS, permeabilized with 0.5% Tween 20 in PBS for 5 min, and then incubated with NGS buffer (5% normal goat serum, 0.5% Tween 20, and 0.1% BSA (Sigma) in PBS) for 30 min to reduce nonspecific binding. Primary antibodies CldU (rat anti-BrdU; Novus Biosciences, 1:300) and IdU (mouse anti-BrdU; BD Biosciences, 1:100) were diluted in NGS buffer, added to the slides, and incubated at room temperature in a humid environment for 2 h. Slides were washed with PBS-Tween 20 and then in a high-salt buffer (250 mM NaCl, 0.2% Tween 20, and 0.2% NP-40 in PBS) for 15 min. The samples were incubated in NGS buffer a second time for 20 min, followed by incubation with secondary antibodies (CldU, donkey anti-rat Alexa Fluor 488 [Molecular Probes/Invitrogen, 1:1000]; IdU, goat anti-mouse Alexa Fluor 546 [Molecular Probes/Invitrogen, 1:1000) for 1 h. Finally, slides were washed with PBS-Tween 20, mounted with Dabco antifade mounting media in glycerol (Sigma), and stored at 4°C. Images were visualized by using a Carl Zeiss confocal microscope and images were processed using Zeiss LSM image browser software.

For Protein/nucleotide staining, cells after incubation with 100 μM IdU for 45 min in different conditions were fixed at the indicated times after removal of IdU with 3.7% formaldehyde for 10 min. The cells were washed and incubated with methanol for 15 min at −20°C. Fixed cells were stored in 70% ethanol at 4°C for overnight. At the time of antibody staining, ethanol was removed, and cells were washed twice with PBS and incubated for 1 h with 5% BSA in PBS to block nonspecific binding. After a PBS wash, the cells were incubated for 2 h with antibodies specific to the desired proteins diluted in 1% BSA in PBS. Slides were washed twice with PBS and then incubated with secondary antibodies conjugated with Alexa Fluor 488 for 1 h. After a PBS wash, the cells were again fixed with 4% formaldehyde for 5 min, followed by 10 min incubation with 1.5 N HCl at 37°C to denature the DNA. Cells were washed again, incubated with 0.5% Tween 20 in PBS for 5 min, and incubated with NGS for 20 min. IdU primary antibody (mouse anti-BrdU [BD Biosciences; 1:200) was diluted in blocking buffer and incubated for 2 h in a humid environment. Cells were washed and incubated with anti-mouse conjugated with Alexa Fluor 546 (Molecular Probe, 1:1000) for 1 h, washed, and mounted by using mounting medium. Images were visualized by using a Carl Zeiss confocal microscope and images were processed using Zeiss LSM image browser software.

### Chromatin immunoprecipitation (ChIP)

Cells were cultured overnight at a density of 1 × 10^7^ per 150 mm Petri dish and subjected to either no treatment or treatment with 0.5 μM aphidicolin or HU for 24 h. Chromatin and proteins were cross-linked by incubating cells in 1% formaldehyde for 15 min at room temperature and the reaction was stopped by 10 min incubation with 125 mM glycine. Cells were collected and washed sequentially with solution A (10 mM HEPES [pH7.5], 10 mM EDTA, 0.5 mM EGTA, 0.75% Triton X-100) and solution B (10 mM HEPES [pH7.5], 200 mM NaCl, 1 mM EDTA, 0.5 mM EGTA). The cell pellets were resuspended in 1 ml lysis buffer (25 mM Tris-HCl [pH7.5], 150 mM NaCl, 0.1% SDS, 1% Triton X-100, 0.5% deoxycholate freshly supplemented with protease inhibitor cocktail (Roche) and sonicated on ice by 10s pulses at 25% of maximal power on a sonicator. After centrifugation at 13 000 rpm for 15 min to remove any debris, the supernatant was pre-cleared with protein-G-sepharose/salmon sperm DNA beads at 4°C for 1h. For each immunoprecipitation, 600 μl of the pre-cleared chromatin was incubated overnight at 4°C with 6 μg of antibodies specific for RAD51, RAD51C, XRCC2, XRCC3, RECQ1, γH2AX and 53BP1. A reaction containing an equivalent amount of Goat/rabbit IgG was included as the background control. 10% of the pre-cleared chromatin was taken as input control. Antibody-chromatin complexes were pulled down by adding 50 μl of protein-G-sepharose/salmon sperm DNA beads and incubated for 4 h at 4°C. The beads were washed for 10 min each with the lysis buffer followed by high-salt wash buffer (0.1% SDS, 1% Triton X-100, 2 mM EDTA, 20 mM Tris-HCl [pH 8.1], 500 mM NaCl), LiCl wash buffer (250 mM LiCl, 1% NP-40, 1% deoxycholate, 1 mM EDTA, 10 mM Tris-HCl [pH 8.0]), and TE buffer (10 mM Tris-HCl [pH 8.0], 1 mM EDTA). Finally, DNA was eluted with elution buffer (1% SDS, 100 mM NaHCO3). Elutes were incubated at 65°C for overnight with the addition of 5 M NaCl to a final concentration of 200 mM to reverse the formaldehyde cross-linking and digested at 55°C for 3 h with proteinase K at a final concentration of 50 μg per ml. Following phenol/chloroform extraction and ethanol precipitation, sheared DNA fragments served as template in semi-quantitative PCR analysis. The sequences of the PCR primers are FCR Forward- 5′-TGTTGGAATGTTAACTCTATCCCAT-3′; FCR Reverse 5′ATATCTCATCAAGACCGCTG- CA-3′; FDR Forward- 5′-CAATGGCTTAAGCAGACATGGT-3′; FDR Reverse- 5′-AGTGAA- TGGCATGGCTGGAATG-3′; FRA16D Forward- 5′-TCCTGTGGAAGGGATATTTA-3′; FRA16D Reverse- 5′-CCCCTCATATTCTGCTTCTA-3′; β-actin Forward- 5′-GACGCAGGA- TGGCATGGG-3′ and β-actin Reverse- 5′-ACGCCTCTGGCCGTACCAC-3′.

### DNA fiber spreading

Approximately 5 × 10^5^ cells were plated in each well of a six-well plate. Cells were pulse-labeled with 100 μM IdU and 100 μM CldU before replication stalling with 4 mM HU, as indicated in the sketches. Later, cells were harvested and re-suspended in 50 μl of PBS. Cell suspensions (2.5 μl) were mixed with 7.5 μl of lysis buffer (0.5% sodium dodecyl sulfate, 200 mM Tris-HCl [pH 7.4], 50 mM EDTA). Each mixture was dropped on the top of an uncoated regular glass slide. Slides were inclined at 45° to spread the suspension on the glass. Once dried, DNA spreads were fixed by incubation for 5 min in a 3:1 solution of methanol-acetic acid. The slides were dried and placed in prechilled 70% ethanol at 4°C for at least 1 h or overnight. Slides were then incubated in methanol and washed in PBS. DNA was denatured with 2.5 N HCl for 30 min at 37°C. The slides were rinsed several times in PBS and incubated with the following antibodies: mouse anti-BrdU antibody (BD biosciences, 1:100) and rat anti-CldU (Novus biosciences, 1:300) diluted in 1% BSA. After incubation in a humid chamber for 1 h at 37°C, slides were washed three times, each time for 3 min in PBS containing 0.1% Triton X-100. The slides were incubated with secondary fluorescent antibodies (Alexa anti-mouse 488 [Molecular Probes/Invitrogen, 1:1000] and Alexa anti-rat 546 [Molecular Probes/Invitrogen, 1:1000] diluted in 1% BSA) for 1 h at 37°C. Slides were washed three times for 3 min in PBS-0.1% Triton X-100 and mounted by using Dabco mounting media. Fibers were imaged (Olympus microscope) and analyzed using Image-J software. Statistics were calculated using Graph pad software.

### BrdU incorporation assay

Cells were incubated with 50 μM bromodeoxyuridine (BrdU; Sigma) for 40 min (as indicated in the figure). Cells were fixed in 70% cold ethanol at the indicated times and stored at 4°C. DNA was denatured by using 2 N HCl and 0.5% Triton X-100 and then neutralized with 0.1 M sodium borate (pH 8.5). After two washes with 0.5% Tween 20 and 0.5% bovine serum albumin (BSA) in phosphate-buffered saline (PBS), anti-BrdU antibody (BD biosciences, 1:1000) was added for 2 h followed by anti-mouse secondary antibody conjugated to FITC (Sigma) for 1 h. Later, after two washes with PBS, samples were incubated with RNase-propidium iodide and analyzed on an FACS canto flow cytometer (Becton Dickinson).

### Cell survival assay and metaphase spreads

Cells (500) were seeded onto 100 mm Petri dish in duplicates then treated with various DNA damaging agents at the indicated dose or concentration. Treated cells were either recovered from the genotoxic agents or left alone, and then grown for 10–14 days before staining with crystal voilet. Colonies containing >50 cells were counted as one cell. Percent growth was calculated as (treated cells/untreated cells x100). For metaphase spreads, cells were treated with HU and later incubated with 1 μg/ml colcemid for the last 4 h. Cells were then harvested and treated with hypotonic solution (75 mM KCl) for 12 min, washed with chilled fixative (methanol/acetic acid 1:1), and left overnight at 4°C. Cells were later dropped onto a chilled glass slide, air-dried and stained with 5% aqueous Giemsa. For each case, 50 metaphase plates were scored.

## RESULTS

### RAD51 paralogs are enriched onto the S-phase chromatin and interact with nascent DNA at stalled/collapsed replication forks

**Figure 1.**
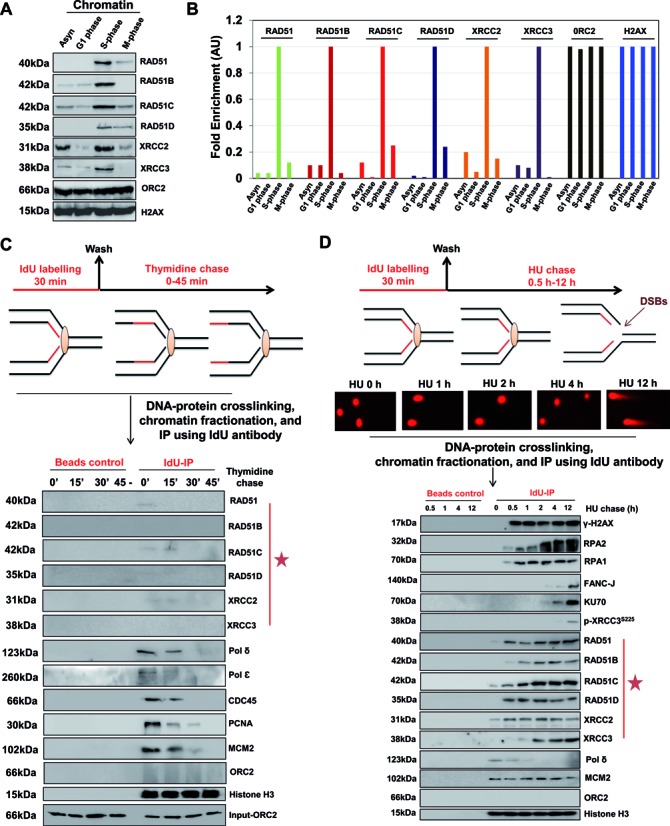
RAD51 paralogs associate with S-phase chromatin and bind to nascent DNA at stalled/collapsed replication forks. (**A**) U2OS cells were synchronized by serum starvation (G0/G1), thymidine-aphidicolin block (S) and nocodazole (G2/M). Chromatin extracts were prepared at indicated cell cycle stages and analyzed for RAD51 and RAD51 paralogs. (**B**) Quantification of western blots from two independent experiments showing fold enrichment of indicated proteins at the chromatin at indicated cell cycle stages. (**C**) Experimental design for nascent strand pull-down of active replication forks. HeLa cells were labeled with IdU for 30 min, followed by a *chase* into *thymidine*-containing medium for the indicated times. Cells were cross-linked, and the chromatin fraction was isolated (input) and subjected to IP using anti-IdU antibody (Idu-IP) or only beads (Beads control). Fractions were probed for indicated proteins with ORC2 as control (input and negative control). (**D**) Experimental design for nascent strand pull-down of HU-mediated stalled/collapsed replication forks. HeLa cells were labeled with IdU for 30 min, followed by a *chase* into 1 mM HU-containing medium for the indicated times. Representative neutral comet images of HeLa cells treated with 1 mM HU for the indicated times are shown. Cells were cross-linked, and the chromatin fraction was isolated and subjected to IP using anti-IdU antibody (Idu-IP). Fractions were probed for indicated proteins with Histone H3 as loading control.

To test whether RAD51 paralogs participate in the repair of spontaneously arising replication stress, we measured spontaneous chromatin binding of RAD51 and RAD51 paralogs at various phases of the cell cycle. U2OS cells were synchronized in G0/G1, S and G2/M (Supplementary Figure S1B), and whole cell extracts as well as chromatin samples (Figure 1A, Supplementary Figure S1A and B) were analyzed for the abundance of RAD51 and RAD51 paralogs. Although the expression levels of RAD51 paralogs were fairly constant at different phases of the cell cycle (Supplementary Figure S1B), their chromatin association was specifically enriched in the S-phase (Figure 1A and B). To validate this further, while avoiding thymidine mediated replicative damage, U2OS cells were synchronized in G2/M by nocodazole, recovered at different phases and spontaneous chromatin association of RAD51 and RAD51 paralogs was measured. Consistent with earlier data, RAD51 and RAD51 paralogs were enriched on S-phase chromatin (Supplementary Figure S1C). Next, we monitored the kinetics of chromatin loading of RAD51 paralogs and replication factors during G1 to S-phase transition in undamaged conditions (Supplementary Figure S2A). Chromatin loading of RAD51 paralogs was slower than the assembly of other essential DNA replication factors (Supplementary Figure S2A). Moreover, association of RAD51 paralogs with S-phase chromatin was impaired by inhibition of CDKs (Supplementary Figure S2B).

Next, we asked whether S-phase chromatin loading of RAD51 paralogs correlates with their association at nascent strands during the course of active replication. To this end, we used the thymidine analog 5-Iodo-2′-deoxyuridine (IdU) to label ongoing DNA synthesis, and later immunoprecipitated it from cross-linked chromatin to analyze replication machinery (Supplementary Figure S3A). IdU-IP was combined with thymidine chase for increasing time to monitor DNA-associated proteins at increasing distances from the moving fork (Figure 1C). Constant histone H3 levels served as control, and as expected, the association of MCM2, PCNA, CDC45, Pol ϵ and Pol δ with the IdU-labeled fragment declined within 15–30 min of chase time (Figure 1C), indicating the progression of replication fork. However, the amount of RAD51 and RAD51 paralogs detected at nascent DNA was fairly insignificant at all times (Figure 1C), suggesting that RAD51 paralogs are not a part of active replisome at the nascent DNA strands. To validate this observation, we monitored DNA replication by DNA fiber technique in Chinese hamster cells deficient for RAD51C, XRCC2 and XRCC3 in comparison with parental WT cells in unperturbed S-phase with IdU followed by 5-Chloro-2′-deoxyuridine (CldU) (Supplementary Figure S3B). Indeed, WT and RAD51 paralog deficient cells exhibited almost identical fork velocity with an average fork progression rate of 1–1.3 kb/min (Supplementary Figure S3B). After confirming the dispensable nature of RAD51 paralogs in unperturbed DNA replication, we performed IdU-IPs in conditions of perturbed replication with increasing length of hydroxyurea (HU) (Figure 1D). It has been previously shown that during acute HU induced stress, stalled forks are stable for several hours and viable to resume replication (44,51). In contrast, chronic exposure to HU leads to forks collapse and generation of DSBs (44,51–53). Indeed using neutral comet assay for specific detection of DSBs, we observed that HU-stalled replication forks are devoid of DSBs up to 4 h but later collapse into DSBs at 12 h (Figure 1D). Notably, our analysis with IdU-IP suggested the loading of RAD51 and RAD51 paralogs at nascent DNA as early as 0.5 h to 2 h of HU treatment with a concomitant decrease in polymerase, suggestive of replication fork inactivation with increasing HU duration (Figure 1D). Further increase in HU length for up to 12 h led to the replication fork collapse as indicated by DSB markers FANCJ, KU70 and phosphorylated XRCC3 (Figure 1D). The presence of γ-H2AX and RPA1 at the fork was evident from 0.5 h and remained constant for up to 12 h of analysis, indicative of fork uncoupling and early ATR, and later ATM activation during the entire course of HU treatment (Figure 1D). The levels of MCM2 and Histone-H3 remained constant for up to 12 h (Figure 1D). Together these data suggest that under replicative stress, RAD51 and RAD51 paralogs are enriched at replication sites both before and after the generation of breaks at the fork.

Consistent with previous studies (25,26,42,54,55), we observed that RAD51 paralogs deficient cells exhibit marked sensitivity towards range of replication poisons such as HU, aphidicolin and camptothecin (CPT) (Supplementary Figure S3C). We incubated RAD51C, XRCC2 and XRCC3 deficient cells continuously with mild doses of CPT, HU and aphidicolin, and observed that RAD51C and XRCC3 cells were hypersensitive toward these agents compared to the mild sensitivity of XRCC2 deficient cells (Supplementary Figure S3C). Next, we asked whether localization of RAD51 paralogs to nascent strands at stalled forks is a general phenomenon to variety of replication poisons. To this end, we carried out nascent stand isolation after UV irradiation. UV exposure leads to template damage and, in a fraction of repair cases, leading strand damage results in the generation of daughter strand gaps (DSGs) after re-priming ahead of the lesion (17,56). In order to understand the role of RAD51 paralogs in the repair of DSGs behind the fork, we performed IdU-IP after IdU labeling followed by UV-C irradiation, and later thymidine chase for up to 60 min (See scheme Supplementary Figure S4A). Our data revealed that RAD51 and RAD51 paralogs remain at damaged site even after the movement of replisome ahead of lesion as assessed by the disappearance of MCM2 and replicative polymerases with time (Supplementary Figure S4A). Kinetics of RAD51C recruitment was found to be faster than RAD51, XRCC2 and XRCC3 (Supplementary Figure S4A), suggesting a more critical role of RAD51C in fork maintenance.

### RAD51 paralogs suppress spontaneously arising replication-based lesions and localize to common fragile sites during replicative stress

**Figure 2.**
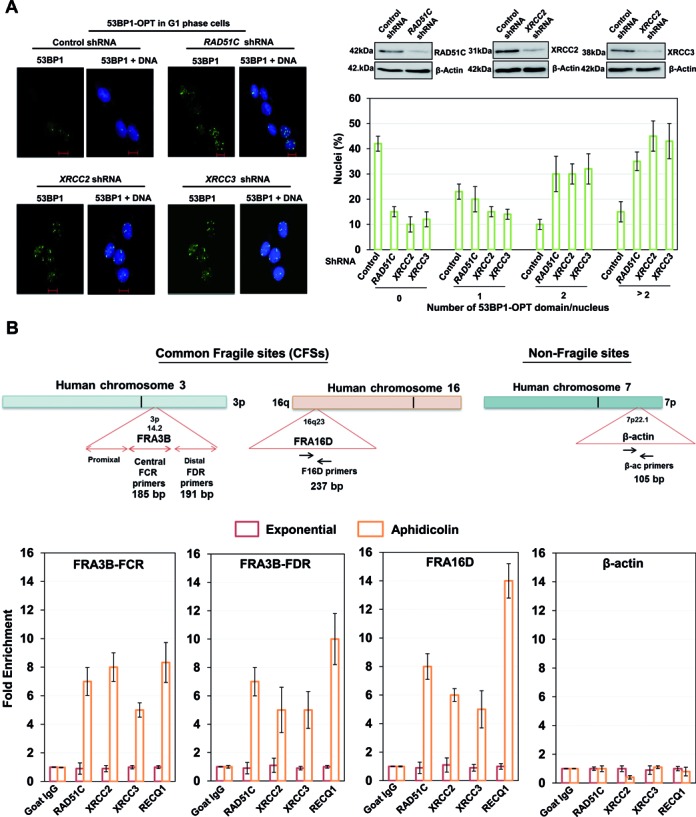
RAD51 paralogs localize to Common Fragile Sites (CFSs) upon replicative stress and suppress replication associated lesions. (**A**) Representative images and quantification of G1 phase synchronized U2OS control and RAD51C, XRCC2 and XRCC3 depleted cells, fixed and stained with 53BP1 antibody to visualize OPT domains (53BP1-green and DNA-blue). At least 100 G1 cells were counted for each experiment. Blots for depletion of RAD51C, XRCC2 and XRCC3 have been shown. (**B**) Genomic organization of the FRA3B, FRA16D and β-Actin (ACTB) region. Primer sets of distal (FDR) and central (FCR) region within the FRA3B locus, FRA16D locus and β-Actin locus are indicated. Quantification of cross-linked FRA3B-FCR, FRA3B-FDR, FRA16D and β-Actin loci chromatin immunoprecipitated from HeLa cells using indicated antibodies. RECQ1 antibody was used as a positive control for FRA3B and FRA16D enrichment. Fold enrichment over goat IgG was determined and is shown for each primer pair for the ChIP. Results are expressed as mean ± SD for at least three independent experiments.

Since RAD51 paralogs were found to be specifically enriched on S-phase chromatin but did not strongly associate with unperturbed replication forks, we asked whether the observed S-phase enrichment was owed solely to their binding to sites of spontaneous replication stress in cells. Recently, it has been shown that a class of DNA lesions caused by replication stress is transmitted to daughter cells and protected from erosion by 53BP1-nuclear bodies (53BP1-OPT domains) (57,58). These structures persist in G1-phase of the cell cycle but are resolved in the next S-phase. To test the role of RAD51 paralogs in spontaneous replication stress, we examined whether RAD51 paralog deficient cells display increased 53BP1-OPT domains by examining 53BP1 focus formation exclusively in unperturbed synchronized G0/G1 cells. Strikingly, in RAD51C, XRCC2 and XRCC3 depleted cells, the number of 53BP1 foci was significantly higher than in control cells (Figure 2A), indicating that RAD51 paralogs prevent under-replication of genomic regions that are intrinsically hard to replicate. Similar results were obtained in hamster cells (data not shown).

Replicative stress particularly affects genomic loci where replication fork progression is slow or problematic (59–63). Having observed the loading of RAD51 paralogs at nascent strands of stalled forks (Figure 1D), we examined whether RAD51 paralogs are recruited to common fragile sites (CFS) after aphidicolin treatment which introduces replication fork stalling at fragile sites (62). We tested recruitment of RAD51 paralogs to stalled replication forks at the FHIT region in the aphidicolin-sensitive fragile site FRA3B. HeLa cells were either untreated or treated with 0.5 μM aphidicolin for 24 h. The cross-linked chromatin prepared from each condition was then processed for ChIP by using either control IgG or RAD51C, XRCC2 and XRCC3 specific antibodies. RECQ1 that binds to FHIT region of FRA3B served as positive control (64). To determine whether RAD51 paralogs occupy FRA3B locus, primers specific to two separate regions in FRA3B fragile locus including the distal and central aphidicolin induced breakpoint clusters (FDR and FCR) located within intron 4 of the FHIT gene were used (Figure 2B). As shown in Figure 2B and Supplementary Figure S4B, RAD51 paralogs or RECQ1 did not bind to the FRA3B locus in untreated cells but treatment with aphidicolin induced their significant enrichment at FRA3B-FDR and -FCR locus. To rule out the possibility of non-specific enrichment, β-actin locus was taken as a negative control (Figure 2B). ChIP experiments after HU treatment (1 mM, 24 h) revealed nearly similar results (Supplementary Figure S4C). To ascertain preferential binding of RAD51 paralogs to CFS, we analyzed loading of RAD51 paralogs to FRA16D, the second most active and aphidicolin-sensitive fragile site in the human genome (Figure 2B), and observed significant enrichment of RAD51 paralogs at the locus upon aphidicolin treatment (Figure 2B). This enrichment of RAD51 paralogs was dependent on CDKs, as inhibition of CDKs completely abrogated the recruitment on CFSs (Supplementary Figure S5A), validating our previous result that only processed structures are occupied by RAD51 paralogs upon replication stress.

RAD51 has been shown to associate with S-phase chromatin independent of BRCA2 (65). To test the role of RAD51 paralogs in loading of RAD51, we examined RAD51 foci formation in S-phase in RAD51 paralog and BRCA2 deficient cells. It was found that RAD51 foci formation in S-phase synchronized cells was abrogated in the absence of RAD51C and XRCC2 but not in XRCC3 and BRCA2 mutant cells (Supplementary Figure S5B). Interestingly, in addition to RAD51 paralogs, RAD51 also localized to the FRA3B site in a CDK dependent manner (Supplementary Figure S5A and B), and this recruitment was affected in RAD51 paralog deficient hamster cells, and with depletion of RAD51 paralogs in human cells (Supplementary Figure S6B). RAD51 paralogs are known to assemble in two stable complexes, BCDX2 and CX3 (66,67). To understand the functions of different complexes of RAD51 paralogs and their independent roles in recruitment of RAD51 at replication forks, we studied the loading of RAD51 and RAD51 paralogs at the nascent strand from the hamster cells deficient in RAD51C (CL-V4B), XRCC2 (irs1) and XRCC3 (irs1SF). In XRCC3 deficient cells, RAD51 and all other RAD51 paralogs bound to nascent DNA under stalled replication conditions (Supplementary Figure S6A). Interestingly, nascent DNA from RAD51C deficient cells showed reduced levels of RAD51B, XRCC3 and RAD51, while loading of RAD51D and XRCC2 remained unaffected (Supplementary Figure S6A). Furthermore, RAD51B, RAD51C and XRCC3 showed nascent strand interaction in XRCC2 deficient cells, while RAD51 and RAD51D loading was severely impaired (Supplementary Figure S6A), suggesting that RAD51 paralogs can get recruited at the stalled forks in three different complexes. These results favor the important role of RAD51C and XRCC2 in RAD51 loading and stabilization at the fork, and XRCC3 might be involved in stabilizing or controlling the loaded form of RAD51.

### RAD51 paralogs suppress breakage of stalled forks

**Figure 3.**
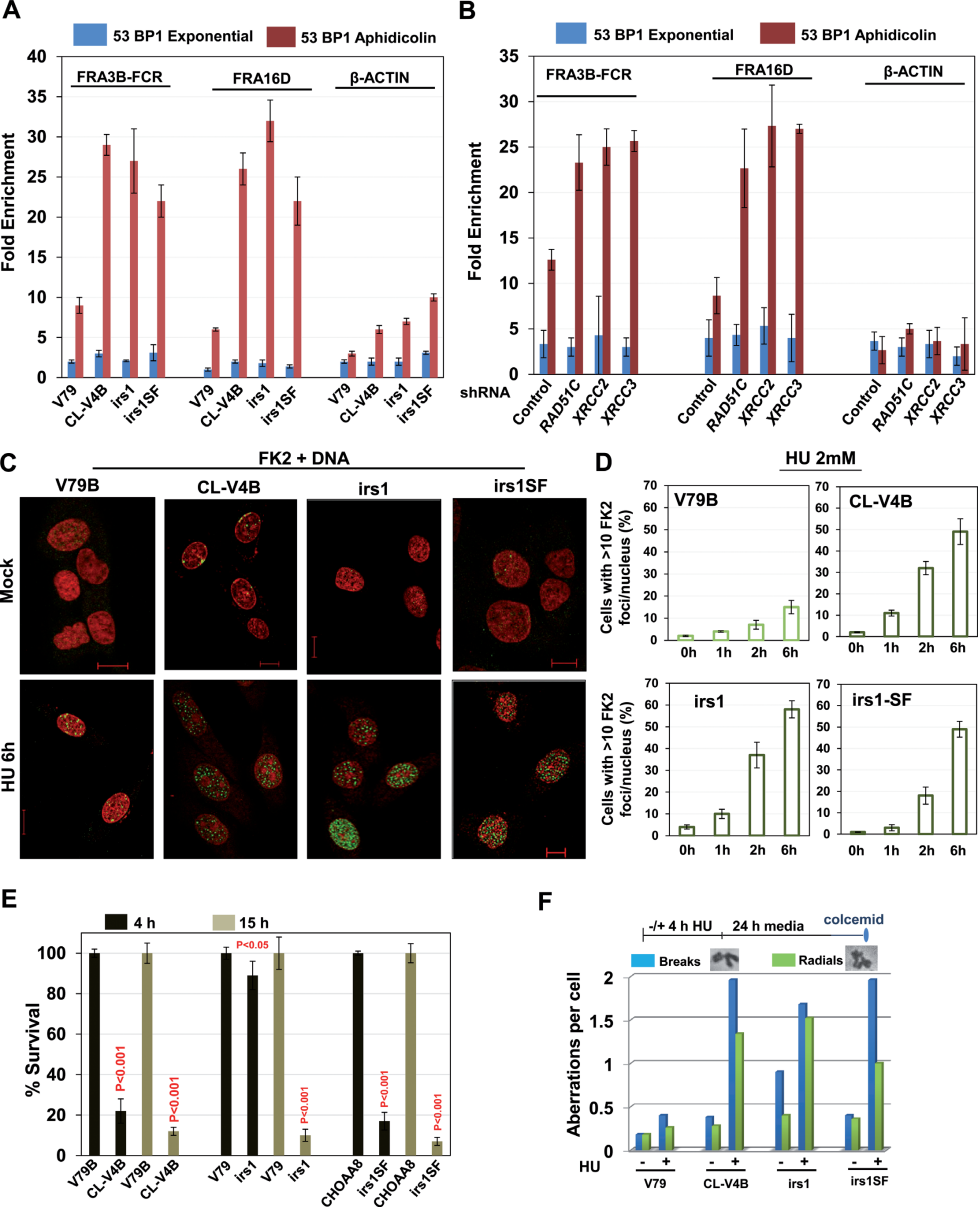
Cells deficient in RAD51 paralogs undergo increased replication fork collapse following replicative stress. Fold enrichment of 53BP1 at FRA3B-FCR and FRA16D loci from RAD51 paralog deficient hamster cells (**A**) or HeLa cells treated with shRNAs for RAD51 paralogs (**B**). Cells were treated with aphidicolin (0.5 μM) for 24 h, and analyzed by ChIP. Data are mean ± SD (*n* = 3). β-actin locus was included as a negative control. (**C**) Representative images of HU-induced ubiquitin foci marked by FK2 antibody in the indicated cells (FK2-green and DNA-red). Scale bar 10 μm. (**D**) Quantitative analysis of the FK2-foci/nucleus in the indicated cells at indicated times of 2 mM HU treatment for 2 h. Bar graph represents average of two independent experiments (±SD). (**E**) Cellular sensitivity of RAD51C, XRCC2 and XRCC3 deficient cells to HU exposure as measured by colony formation assay. Indicated cells were treated with 1 mM HU for 4 or 15 h, and later released in to fresh medium for 10–14 days and cell survival was determined. Results shown are mean ± SD of two independent experiments. *P*-values are obtained for paralog deficient cells in comparison with parental cells. (**F**) Quantification of chromosomal aberrations in the form of chromatid breaks and radial chromosomes in RAD51 paralog deficient cells. (CL-V4B- RAD51C^−/−^, irs1- XRCC2^−/−^, irs1SF- XRCC3^−/−^, and V79B, V79 and CHO-AA8 are parental cell lines for CL-V4B, irs1 and irs1SF respectively).

Unattended replication forks undergo collapse, leading to generation of DSBs at the forks (68). Hence we tested the presence of DNA damage markers at CFSs in paralog deficient hamster cells or after depletion of RAD51 paralogs in human cells. Indeed, analysis of cells exposed to a low dose of aphidicolin followed by ChIP and semi-quantitative PCR revealed an enhanced enrichment of 53BP1 and γ-H2AX at FRA16D and FRA3B loci after genetic ablation of RAD51 paralogs (Figure 3A, B and Supplementary Figure S7A). Generation of DSBs at the forks causes a shift in DNA damage response from ATR kinase to ATM (51,68), which then orchestrates an ubiquitin-dependent sequence of events in response mediated by the RNF8 and RNF168 ubiquitin ligases, which produce ubiquitin conjugates at DSB sites (69,70). These are readily detected by immunofluorescence and thus serve as a dependable marker for DSB formation after fork collapse. We therefore examined the appearance of ubiquitin foci marked by the monoclonal antibody FK2 in HU-treated parental V79B and paralogs deficient cells (Figure 3C). Indeed, with extended HU treatment, irs1, CL-V4B and irs1-SF cells accumulated substantially more FK2 foci than control V79B cells (Figure 3C and D). Surprisingly, the extent of fork collapse measured by FK2 straining revealed slower kinetics of DSB generation in irs1-SF cells compared to irs1 and CL-V4B cells (Figure 3D). These results indicate that RAD51 paralogs supress replication fork collapse, and CFSs instability might contribute to the genesis of replicative stress observed in the RAD51 paralog deficient cells.

To investigate whether extent of DSB generation following HU treatment correlates with the hypersensitivity after acute versus chronic HU treatment, we incubated V79B and U2OS cells with increasing lengths of HU treatment. Continuous treatment of HU for more than 4 h resulted in DSB generation (Figure 1D and Supplementary Figure S7B). In contrast, acute treatment of HU for 4 h arrested the DNA replication completely (Supplementary Figure S7C), but cells were devoid of any DSBs (Figure 1D and Supplementary Figure S7B). Interestingly, RAD51C and XRCC3 cells but not XRCC2 cells exhibited hypersensitivity when cells were exposed to short pulse of HU (Figure 3E). An earlier report showed that BRCA2 deficient cells are insensitive to continuous exposure to HU (14), and consistently, we found that V-C8 cells do not exhibit sensitivity to 4 h of HU treatment but were hypersensitive toward PARP inhibition (Supplementary Figure S8A). Although V-C8 cells did not exhibit sensitivity to HU, these cells displayed increase in chromosomal aberrations compared to parental V79 cells (Supplementary Figure S8B). Similarly, there was a marked increase in chromosomal aberrations in the form of chromatid breaks and radial structures in RAD51C, XRCC2 and XRCC3 deficient cells after 4 h of HU treatment (Figure 3F), suggesting their role in genome maintenance after replication arrest.

### RAD51C, XRCC2 and XRCC3 suppress extensive nascent strand degradation and ssDNA accumulation at the stalled replication forks

**Figure 4.**
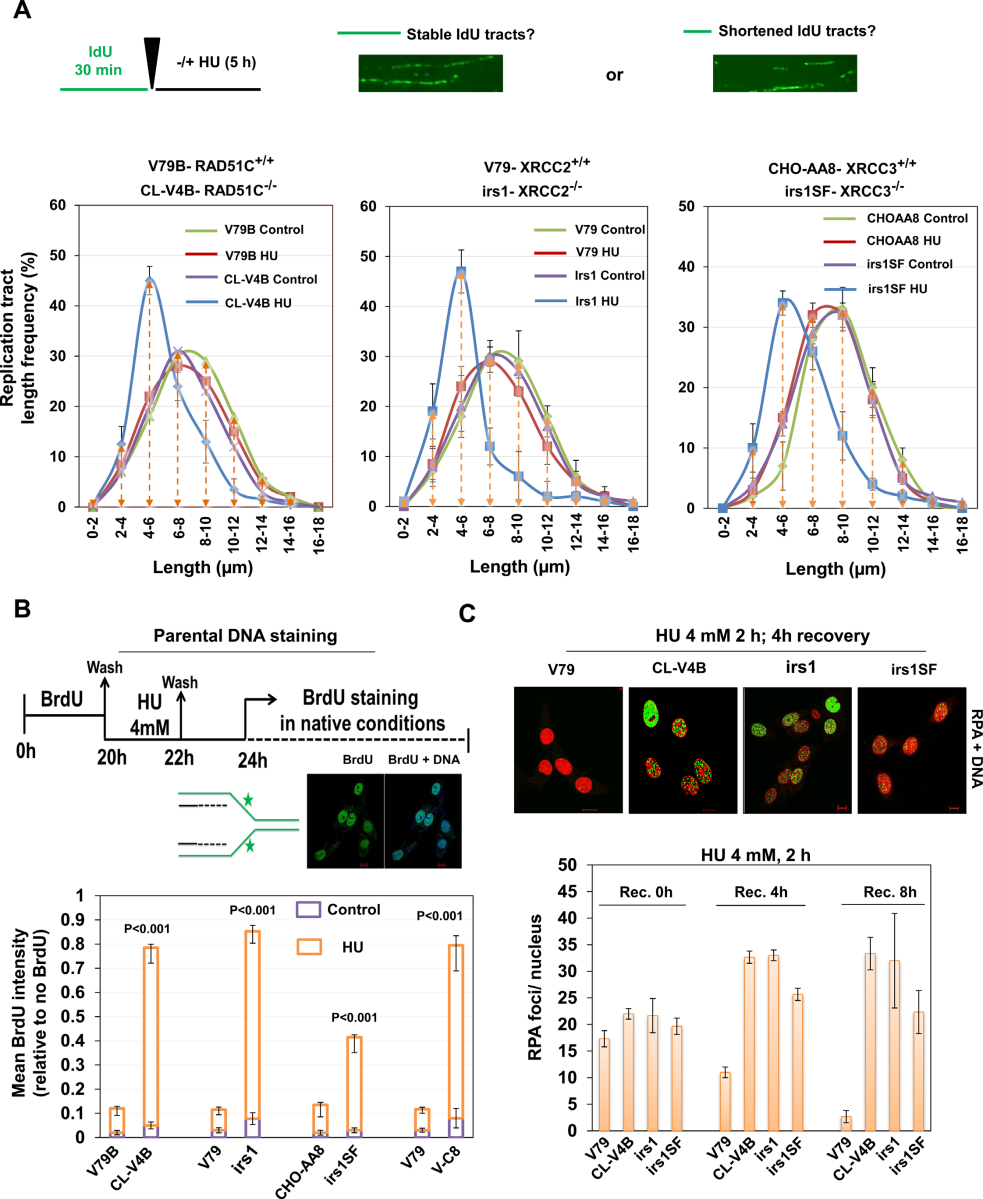
RAD51 paralogs suppress nascent DNA degradation at stalled forks. (**A**) Experimental design of fork protection assay. Length of nascent replication tracts (IdU labeled) were measured by DNA spreading after 5 h of replication stalling with 4 mM HU. Representative DNA fiber image is shown. Measurement of IdU labeled DNA fiber in RAD51 paralog-defective and parental cells. (**B**) Experimental protocol for the parental ssDNA assay. Parental DNA of indicated cells were labeled by the addition of 10 μM BrdU for 20 h followed by a 2 h chase in the presence of 4 mM HU and later recovered in fresh media for 2 h. Cells were fixed and stained with BrdU antibodies without DNA denaturation to selectively detect parental ssDNA. Representative image for BrdU intensity is shown (BrdU-green and DNA-blue). Graph represents the mean BrdU intensity from indicated cells. *P*-values are obtained for paralog deficient cells in comparison with wt V79 cells. (**C**) Representative images of HU-induced RPA foci in the indicated cells after 4 h of recovery. Scale bar 10 μm. Quantitative analysis of the RPA-foci/nucleus in the indicated cells at indicated recovery time points following 4 mM HU treatment for 2 h (RPA-green and DNA-red). Bar graph represents average of three independent experiments (±SD). (CL-V4B- RAD51C^−/−^, irs1- XRCC2^−/−^, irs1SF- XRCC3^−/−^, and V79B, V79 and CHO-AA8 are parental cell lines for CL-V4B, irs1 and irs1SF respectively).

It has been shown that BRCA2, RAD51 and FA proteins stabilize and protect stalled forks independent of their role in DSB repair (14,15,19), thus preventing chromosomal instability. Because RAD51 paralogs are recruited at stalled forks and CFSs, we tested their potential function in fork stability. Nascent replication tracts were IdU-labeled before replication stalling with HU (Figure 4A); the retention of the label after HU treatment serves as a measure for fork stability using DNA fiber spreading (Figure 4A). To test the involvement of RAD51 paralogs in protecting stalled replication forks, we monitored the stability of nascent replication tracts in RAD51C, XRCC2 and XRCC3 defective hamster cells. Replication stalling caused a dramatic shortening of the median IdU tract length in CL-V4B, irs1 and irs1SF cells compared to respective V79B, V79 and CHO-AA8 parental cells (Figure 4A; 7 μm, 6 μm and 9 μm compared to 11 μm, 12 μm and 13 μm, respectively). To directly test whether the nascent strands are resected to expose parental ssDNA at stalled forks in the absence of paralog activity, we developed an assay to selectively detect the appearance of ssDNA at the replication fork. After overnight labeling with BrdU, we released cells into fresh medium for 2 h before treating with HU and performed staining using BrdU antibody which under non-denaturing conditions selectively recognizes ssDNA (Figure 4B). Treatment with HU in V79B, V79 and CHO-AA8 parental cells resulted in little BrdU staining. Conversely, treatment with HU in CL-V4B, irs1 and irs1SF resulted in robust BrdU staining, indicating that the nascent strands are degraded to expose ssDNA on parental strands (Figure 4B). Next, we measured the formation and loss of RPA foci after recovery from HU treatment and observed that increase in RPA foci after HU treatment was reversed in V79 cells but not in RAD51 paralog deficient cells (Figure 4C). RAD51 paralogs defective cells showed ∼3-fold increase in RPA foci per nucleus after 4 h of recovery, which remained constant at 8 h (Figure 4C).

### RAD51C and XRCC2 protect stalled DNA replication forks from extensive processing by MRE11 in a non-epistatic manner to FA-BRCA pathway

**Figure 5.**
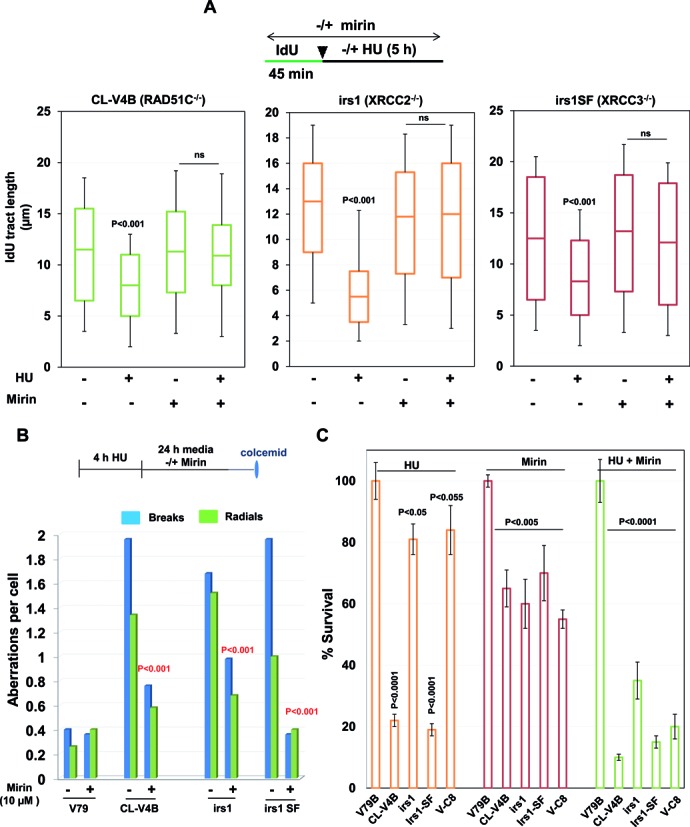
RAD51 paralogs suppress MRE11-mediated extensive fork processing of stalled replication forks. (**A**) Experimental design for analysis of replication fork stability in RAD51 paralogs-deficient cells after chemical inhibition of MRE11 nuclease. IdU labeled DNA fibers were measured in the indicated cells following mock or HU treatment with or without inhibition of MRE11 with Mirin. Bar graphs represent median IdU tract length from 200 analyzed DNA fibers. (**B**) Quantification of chromosomal aberrations in the form of chromatid breaks and radial chromosomes in RAD51 paralog deficient cells with or without inhibition of MRE11. (**C**) Cellular sensitivity of RAD51C, XRCC2, XRCC3 and BRCA2 deficient cells to HU treatment following MRE11 inhibition. Indicated cells were treated with or without 10 μM Mirin, followed by 2 mM HU for 4 h and cell survival was determined. Results shown are mean ± SD of three independent experiments. *P*-values are obtained for paralog deficient cells in comparison with parental cells. (CL-V4B- RAD51C^−/−^, irs1- XRCC2^−/−^, irs1SF- XRCC3^−/−^, V-C8- BRCA2^−/−^ and V79B, V79, and CHO-AA8 are parental cell lines for CL-V4B, irs1 and irs1SF respectively).

FA-BRCA proteins have been implicated to promote replication fork stability in RAD51 dependent pathway (14,15). Consistent with previous reports, BRCA2 deficient V-C8 cells and FANCD2 defective cells exhibited shortening of the median IdU tract length compared to control cells (14) (Supplementary Figure S8C). Given the role of RAD51 paralogs in the recruitment and stabilization of RAD51 after replication fork stalling, we set ourselves to understand the relationship between BRCA2 and RAD51 paralogs for replication fork protection. To this end, we examined replication tract length in V-C8 cells depleted of XRCC2, XRCC3 and RAD51C, and we found that depletion of XRCC2 and RAD51C but not XRCC3 further reduced the tract length in V-C8 cells (Supplementary Figure S9A), suggesting the additive roles of XRCC2 and RAD51C in BRCA2 pathway of fork protection. FANCD2 was shown to protect nascent tract length after HU treatment in an epistatic fashion to BRCA2 and RAD51 (15). We analyzed the effect of FANCD2 depletion onto the nascent tract length after HU treatment in RAD51 paralog deficient and proficient hamster cells. Depletion of FANCD2 in IBR3 and V79 cells resulted in shortening of IdU tracts (Supplementary Figure S8D) which further got aggravated in FANCD2 depleted CL-V4B and irs1 cells (Supplementary Figure S9B). These results suggest that XRCC2 and RAD51C are not epistatic to FA-BRCA pathway, at least in the protection of stalled forks. However, as reported earlier (71,72), we find an epistatic relationship between RAD51 paralogs and FA-BRCA proteins in the repair of DSBs (Supplementary Figure S9C). MRE11 nuclease promotes degradation of stalled replication forks when BRCA2, FANCD2 or RAD51 function is impaired (15,19,47). Indeed, inhibition of MRE11 nuclease with Mirin blocked nascent tract shortening in RAD51 paralog deficient cells (Figure 5A), suggesting a similar mode of fork protection by RAD51 paralogs and FA-BRCA proteins in an additive fashion. Strikingly, although MRE11 inhibition rescued chromosomal aberrations upon HU treatment in RAD51 paralog deficient cells (Figure 5B), it failed to rescue the cell sensitivity in paralog and BRCA2 deficient cells (Figure 5C). These data reveal that cells defective for paralogs or FA-BRCA proteins survive with MRE11 dependent pathway to overcome permanent replication associated damages, however at the cost of compromising genome integrity.

### Extensive fork processing overlaps with loss of replisome components and defect in replication restart in RAD51C defective cells

**Figure 6.**
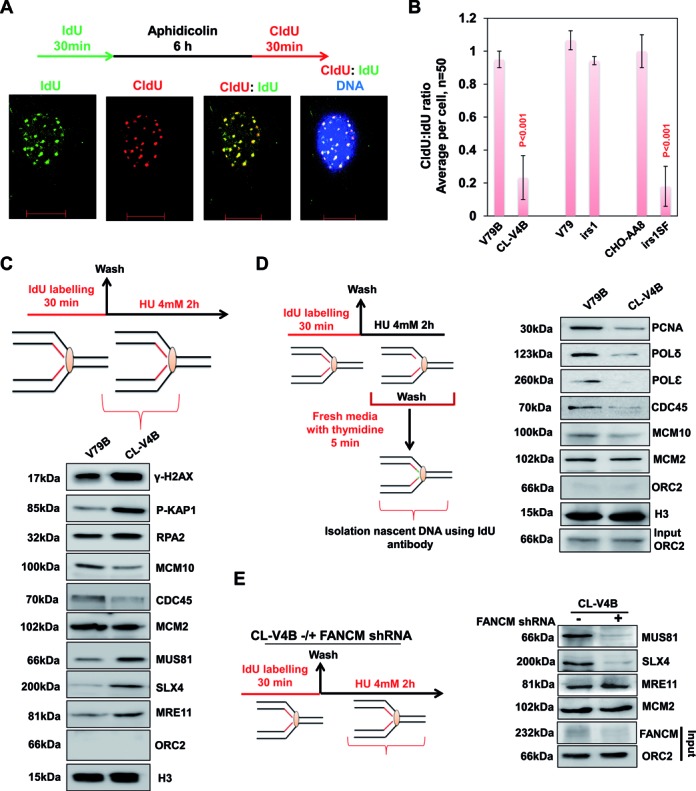
RAD51C deficiency leads to loss of replisome components from stalled forks and impairs restart. (**A**) Representative image for replication restart after aphidicolin treatment. (**B**) Quantification of IF data in indicated RAD51 paralog defective cells. Data are mean ± SD (*n* = 3). Quantification of replication restart after aphidicolin treatment in XRCC3 depleted irs1 and V-C8 cells (BRCA2^−/−^). *P*-values are obtained for paralog or BRCA2 deficient cells compared to respective parental cells. (**C**) Experimental design for nascent strand pull-down of HU-mediated stalled replication forks. wt V79B or RAD51C^−/-^ CL-V4B cells were labeled with IdU for 30 min, followed by release into 4 mM HU-containing medium for 2 h. Cells were cross-linked, and the chromatin fraction was isolated and subjected to IP using anti-IdU antibody. Fractions were probed for indicated proteins with Histone H3 as loading control. (**D**) Experimental design for monitoring fork recoupling after recovery from HU. HeLa cells were labeled with IdU for 30 min, followed by 4 mM HU treatment for 2 h. Later cells were released into *thymidine*-containing medium for 5 min, cross-linked, and the chromatin fraction was isolated (input) and subjected to IP using anti-IdU antibody. Fractions were probed for indicated proteins with ORC2 as control (input and negative control). (**E**) FANCM deficiency rescues excessive processing of stalled forks in RAD51 paralog deficient cells. RAD51C deficient CL-V4B cells treated with FANCM shRNA were labeled with IdU, followed by 4 mM HU treatment for 2 h. Cells were cross-linked, and the chromatin fraction was isolated (input) and subjected to IP using anti-IdU antibody. Fractions were probed for indicated proteins.

Previous studies suggest that prolonged replication stress leads to decrease in the association of replication factors with chromatin and elevated degradation of specific replisome components (51,52). Indeed, a more recent study has identified novel pathways that lead to degradation of replication factors in response to replisome instability (52), thus rendering the forks incapable of resuming replication upon release from the replicative stress. To test directly whether RAD51 paralog deficiency affects replication restart, we tested the ability of RAD51 paralog and BRCA2 deficient cells to recover from replication stalling by visualizing DNA replication by sequential incorporation of halogenated deoxyuridine derivatives. Thus, we marked replicating cells by pulse labeling with 5‐iodo‐2‐deoxyuridine (IdU), arrested them with aphidicolin for 6 h, and then resumed replication by removing the drug and monitored the replication with 5‐chloro‐2‐deoxyuridine (CldU) (Figure 6A). WT and XRCC2 deficient cells exhibited complete recovery of forks after aphidicolin treatment, but both RAD51C and XRCC3 cells displayed severe replication restart defects (Figure 6B). Collectively, these data suggest that both RAD51C and XRCC3 are critical for fork restart after aphidicolin treatment. To further understand the activation of RAD51 paralogs at sites of stalled replication forks in distinct stalled and recovering conditions, we tested whether the RAD51 paralogs localize to sites of DNA replication. Cells were pulse-labeled with IdU for 30 min in the presence of HU or during recovery. Consistent with their observed role in replication restart, RAD51C and XRCC3 foci in the HU-treated cells coincided with the replication foci marked by IdU at both stalled and recovered forks (Supplementary Figure S10B and C). In contrast, XRCC2 foci coincided only with stalled replication forks (Supplementary Figure S10A). To further explore the distinct roles of XRCC2, RAD51C and XRCC3 in replication recovery, we examined the ability of RAD51 paralog deficient cells to recover from HU‐induced cell‐cycle arrest. Since HU treatment is expected to primarily affect S-phase cells, we tested whether RAD51 paralogs depleted S-phase cells labeled with BrdU before replicative stress could recover and complete DNA replication (Supplementary Figure S10D). Our analysis by flow cytometry suggested that in control cells the replicating population progressed through S and G2/M phase and entered G1-phase after 6 h of recovery and by 24 h, labeled cells entered into next S-phase (Supplementary Figure S10D). Interestingly, RAD51C and XRCC3 depleted cells failed to progress through S-phase for up to 18 h of recovery (Supplementary Figure S10D). XRCC2 depleted and VC-8 cells showed continued progression through S-phase and entered G2/M by 18 h. However, co-depletion of XRCC3 with XRCC2 or in VC-8 cells abolished the S-phase progression (Supplementary Figure S10D), further confirming its role in replication recovery.

To determine whether defect in replication fork restart in RAD51 paralogs deficient cells correlates with the dissociation of replisome components from chromatin, IdU-IPs were performed from V79B and CL-V4B cells after 2 h of HU treatment and examined for the abundance of a variety of replication factors directly at the stalled forks (See scheme Figure 6C). Notably, with the exception of MCM2, which is associated with chromatin in excess, a reduced abundance of several replication factors was observed in RAD51C deficient cells after replication recovery (Figure 6C). The levels of MCM10 and CDC45 at the fork in CL-V4B cells were consistently lower than those observed in control cells (Figure 6C). In accordance with increased rates of fork collapse and end resection at the replication site, H2AX and KAP1 phosphorylation were elevated in CL-V4B cells with the abundance of RPA2, MRE11, SLX4 and MUS81 on nascent stands (Figure 6C), indicating a concomitant increase in both DSBs and ssDNA by the action of nucleases which might result in fork collapse and hence its inactivation. These data clearly demonstrate that the association of several replication factors at the fork is compromized upon replication fork stalling in the absence of RAD51C. To validate further, we isolated nascent strand and monitored fork re-coupling after release from HU block into thymidine (see scheme Figure 6D). Strikingly, release from HU stress resulted in fork recoupling and restart in V79B cells, marked by the association of PCNA, replicative polymerases, CDC45 and MCM10 from the previously stalled forks (Figure 6D). Although there was no change in the level of MCM2, nascent strands from CL-V4B cells showed a severe defect in reloading replication machinery to resume DNA synthesis (Figure 6D). In corroboration with the above results, MUS81 and SLX4 onto the fragile sites were also prominent in CL-V4B, irs1 and irs1SF cells as revealed by ChIP analysis (Supplementary Figure S9D and E).

Elevated levels of MUS 81 and SLX4 at the fork in RAD51C mutant cells points toward excessive fork processing either by fork reversal or just fork degradation. Stalled forks are remodeled and perhaps undergo restart by different mechanisms (48,73,74). Recently, FANCM was identified to promote fork stability and resumption of DNA synthesis after replication pause (75,76). Interestingly, FANCM also has fork remodeling activities (73). Hence, we investigated whether the presence of excessive MUS81 and SLX4 can be reversed by the depletion of FANCM in RAD51C deficient cells. Indeed, nascent strands isolated after HU stress from FANCM depleted CL-V4B cells showed significant reduction in both MUS81 and SLX4 compared to those from CL-V4B control shRNA cells (Figure 6E). Although FANCM depletion resulted in reduced loading of MUS81 and SLX4 at the nascent strands in CL-V4B cells, these cells failed to resume DNA synthesis from the stalled site (data not shown). Notably, level of MRE11 remained high irrespective of FANCM status in CL-V4B cells (Figure 6E). Consistent with this, depletion of FANCM but not WRN inhibition in CL-V4B cells showed remarkable decrease in MUS81 enrichment from FRA3B (Supplementary Figure S9F) and FRA16D sites (data not shown). These results suggest that fork remodeling which is critical for replication restart is controlled by RAD51 paralogs.

### Replication fork protection and restart by RAD51 paralogs require functional Walker motif in a disparate manner

**Figure 7.**
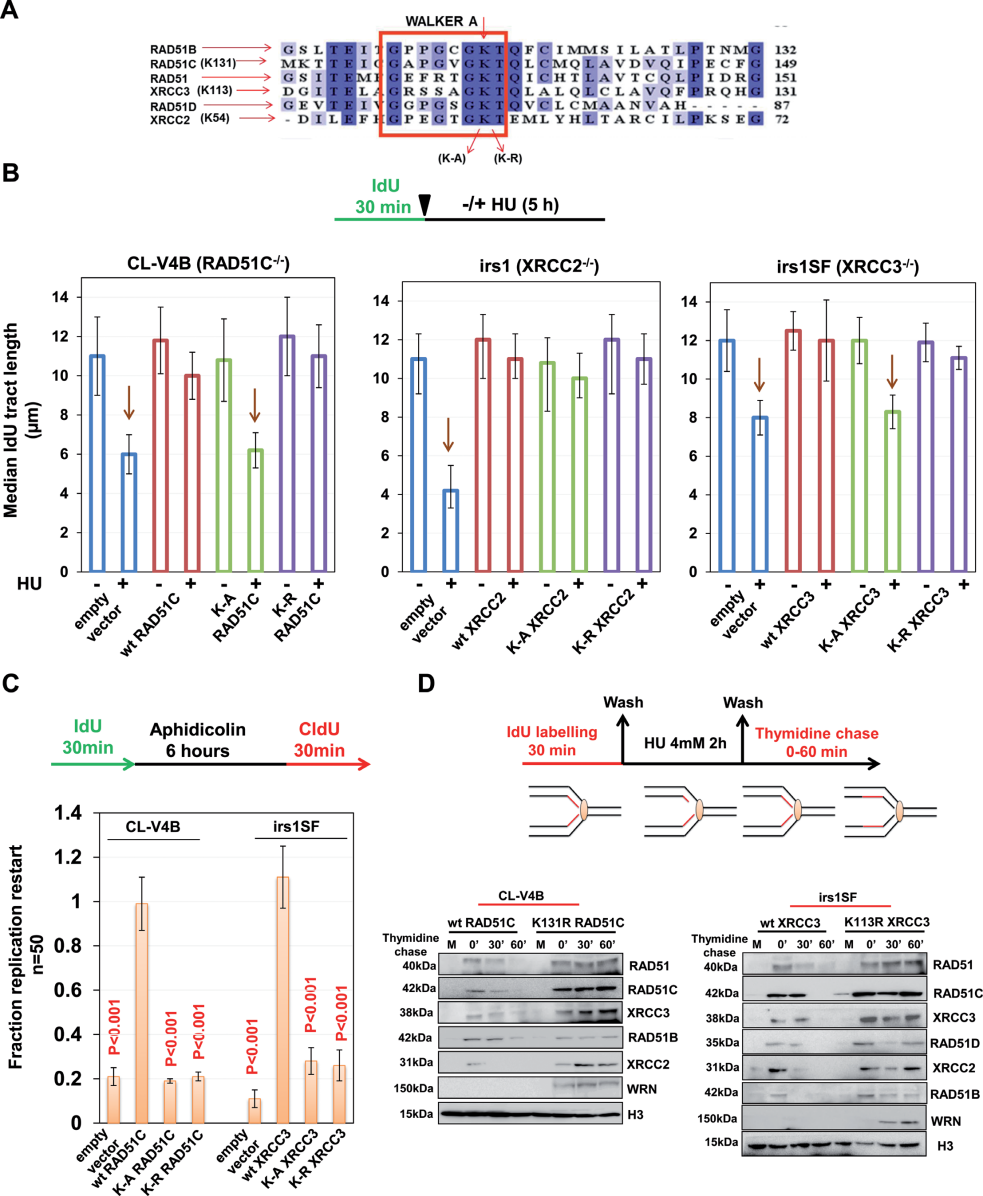
RAD51 paralogs promote replication restart in an ATP hydrolysis dependent manner. (**A**) Sequence alignment of human RAD51 paralogs with the conserved Walker A motif shown in the box. The lysine residue critical for ATP binding is highlighted. (**B**) Experimental design for analysis of replication fork stability in RAD51 paralogs Walker A mutants. Measurement of preformed IdU tract lengths in Walker A mutants of RAD51C, XRCC2 and XRCC3 expressed in CL-V4B, irs1 and irs1SF cells respectively compared to their wt protein. Bar graphs represent median IdU tract length from 100 DNA fibers. (**C**) Quantification of replication restart after aphidicolin treatment in CL-V4B and irs1SF cells after overexpression of wt, KA and KR R51C or XRCC3 respectively as studied by co-localization of IdU and CldU foci. Data are mean ± SD (*n* = 3). *P*-values are obtained in comparison with empty vector cells. (**D**) Experimental design to monitor kinetics of RAD51C and XRCC3 KR mutants at stalled replication forks after release from HU. CL-V4B/irs1SF cells complemented with wt or KR RAD51C/XRCC3 were labeled with IdU for 30 min, followed by 4 mM HU treatment for 2 h and later chased in thymidine-containing medium for indicated times. Cells were cross-linked, and the chromatin fraction was isolated and subjected to IP using anti-IdU antibody. Fractions were probed for indicated proteins. (CL-V4B- RAD51C^−/−^, irs1- XRCC2^−/−^, irs1SF- XRCC3^−/−^, V-C8- BRCA2^−/−^ and V79B, V79, and CHO-AA8 are parental cell lines for CL-V4B, irs1 and irs1SF respectively).

RAD51 paralogs possess Walker A and B motif for ATP binding and hydrolysis (Figure 7A) (77); however its role in genome maintenance is not understood. Given the role of RAD51C, XRCC2 and XRCC3 in replication fork protection, we asked whether the ATP binding and hydrolysis activity of paralogs is required to stabilize stalled replication forks in response to HU treatment. To test this, we expressed human RAD51C, XRCC2 and XRCC3 with point mutations in the Walker A motif (K-A and K-R) in CL-V4B, irs1 and irs1SF cells, respectively. Strikingly, our results from DNA fiber suggest that only ATP binding but not hydrolysis was critical for replication fork stabilization by RAD51C and XRCC3 (Figure 7B). Interestingly, although ATP hydrolysis activity was dispensable for fork protection, it was essential for replication restart by RAD51C and XRCC3 (Figure 7C). To further understand the correlation between ATP hydrolysis and replication restart by RAD51C and XRCC3, we isolated the nascent DNA after HU damage followed by thymidine chase for up to 60 min (see scheme Figure 7D) from WT RAD51C and XRCC3 or from CL-V4B or irs1SF cells that express respective K-R mutants. Strikingly, in contrast to the cells with WT allele, K-R RAD51C expressing CL-V4B cells showed no dissociation of either RAD51 or other paralogs up to 60 min of thymidine chase (Figure 7D). K-R XRCC3 expressing irs1SF cells also failed to dissociate RAD51 and RAD51C from the nascent DNA (Figure 7D). Moreover, there was marked decrease in RAD51B, RAD51D and XRCC2 at 30 min of thymidine chase which again increased at 60 min. This was accompanied by recruitment of WRN, suggesting fork breakdown and DSB generation (Figure 7D). Overall these results reveal that ATP hydrolysis releases RAD51 and paralog complexes from the replication sites during recovery.

### Disease-associated mutants of RAD51C display defective fork maintenance and spontaneously arising replication based lesion

**Figure 8.**
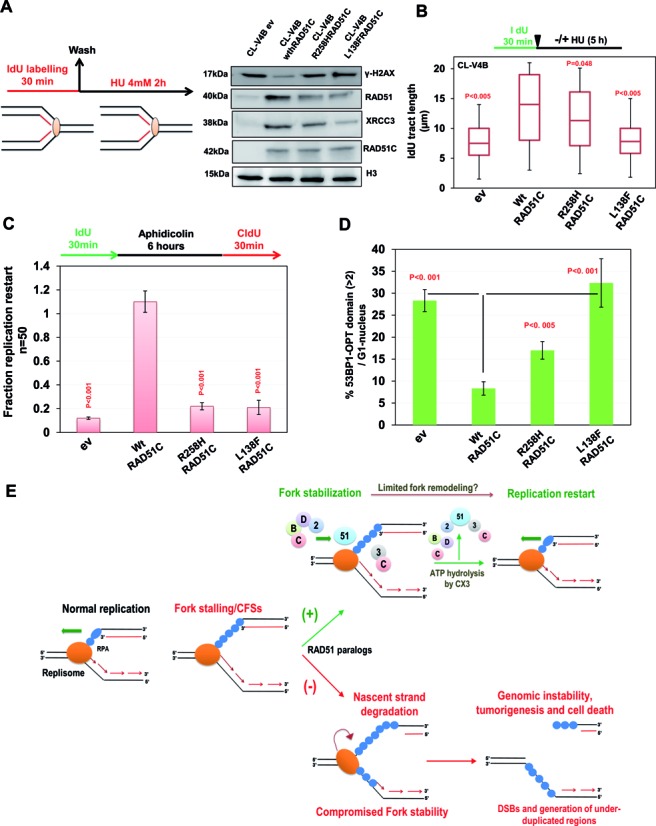
Disease associated mutants of RAD51C are defective in fork maintenance and exhibit spontaneous replication based lesions. (**A**) RAD51C^−/−^ CL-V4B cells complemented with wt, R258H or L138F RAD51C were labeled with IdU for 30 min, followed by 4 mM HU treatment for 2 h. Cells were cross-linked, and the chromatin fraction was isolated and subjected to IP using anti-IdU antibody. Fractions were probed for indicated proteins. (**B**) Measurement of IdU labeled DNA fiber in CL-V4B cells complemented with wt or indicated RAD51C mutants. Bar graph represents median IdU tract lengths of 100 DNA fibers. (**C**) Quantification of replication restart after aphidicolin treatment in CL-V4B cells overexpressing wt or indicated RAD51C mutants as studied by co-localization of IdU and CldU foci. Data are mean ± SD (*n* = 3). *P*-values are obtained in comparison with empty vector cells. (**D**) Quantification of 53BP1 OPT domains in CL-V4B cells overexpressing wt or indicated RAD51C mutants. At least 100 G1 cells were counted for each experiment. (**E**) Model illustrating replisome disassembly after HU induced replication stress in the absence of RAD51 paralogs. Upon replication fork stalling or at common fragile sites, all five RAD51 paralogs bind to nascent DNA strands and protect the fork. BCDX2 and CX3 RAD51 paralog complexes prevent generation of DSBs at stalled replication forks against the action of nucleases. During replication recovery, ATPase activity of RAD51 paralogs promotes the restart of stalled replication forks in an ATP hydrolysis dependent manner. ATP hydrolysis disrupts RAD51 and RAD51 paralogs interaction with the stalled replication forks and limits excessive fork remodeling. Absence of RAD51 paralogs leads to disengagement of replisome components from the fork and allows DNA processing which invites endonuclease mediated cleavage to form DSB, and generation of genome-wide under-duplicated regions and micronucleation.

RAD51C R258H and L138F mutants were identified in FA and breast/ovarian cancer patients, respectively (21,32,33,38). Both of these mutants were previously demonstrated to be functionally impaired for DNA repair by various groups including reports from our lab (25,26,32,33,38,78,79). Previously, we showed that these mutants are defective for direct interaction of RAD51C with RAD51 and other paralogs (26), and consequently elicit faulty HR and intra-S-phase checkpoint response (25,26). Here, we tested the association of RAD51, XRCC2 and XRCC3 with the HU stalled forks in CL-V4B cells expressing WT, R258H or L138F form of RAD51C (Figure 8A). Notably, amount of both the variants of RAD51C was similar to wt protein at the fork, but with elevated levels of γ-H2AX in mutants expressing cells (Figure 8A). Loading of RAD51 at the nascent DNA of stalled forks was markedly impaired in R258H and L138F expressing cells (Figure 8A). Surprisingly, while recruitment of XRCC3 at the fork was severely reduced in the case of L138F expression, it was mildly affected in R258H expressing CL-V4B cells (Figure 8A). Next, we analyzed the fork stability in cells expressing R258H and L138F mutants of RAD51C compared to WT (Figure 8B). While IdU tract length was marginally reduced under R258H expression compared to WT cells, it was degraded significantly in L138F expressing cells similar to empty vector CL-V4B cells (Figure 8B). Next, we studied replication restart in these cells after aphidicolin treatment, and found that both pathological mutations significantly hamper the replication restart capacity of cells (Figure 8C). 53BP1-OPT domain formation was also significantly higher in both R258H and L138F expressing cells as compared to WT cells (Figure 8D). Thus, these results suggest that RAD51C function in replication fork maintenance is compromized in disease causing mutations.

Replication stress, unresolved replication intermediates or under-replicated genome often leads to large deletions and micro-nucleation (80,81). We investigated micro-nucleation in exponentially growing RAD51 paralog deficient cells (Supplementary Figure S10E). Consistent with previous report (45), percentages of cells with micro-nucleation were significantly higher in RAD51C, XRCC2 and XRCC3 deficient cells than with their respective WT cells (Supplementary Figure S10E). This was also true for cells expressing R258H and L138F pathological mutants of RAD51C compared to WT control cells (Supplementary Figure S10F). Together, these results indicate that RAD51 paralogs defect elicits replication associated stress that manifests as 53BP1 nuclear bodies and micro-nucleation in a subset of cells which may contribute to tumorigenesis.

## DISCUSSION

Cells must maintain stable replisomes in order to faithfully duplicate the genome (82). However, replication forks frequently stall during genome duplication specially while progressing through ‘replication slow zones’ (59,63,83). Prolonged fork stalling can lead to fork collapse and can lead to chromosomal breaks and gross chromosomal rearrangements (84). It has been proposed that factors that are required for the HR play crucial roles in the repair of replication based damages (17,85). Indeed, deficiency of RAD51, RAD51 paralogs and FA-BRCA proteins often lead to spontaneous accumulation of replication associated lesions and reduced survival in the presence of replication poisons (86). However, the underlying mechanisms of prevention of replicative stress rather than subsequent repair by recombination factors are still unclear. Therefore, complete understanding of the pathways and the mechanisms required for fork stability during DNA replication is of great importance. Here, we provide experimental evidence to show that similar to FA-BRCA proteins, RAD51 paralogs in different complexes protect stalled replication forks, prevent their collapse into DSBs and also regulate replication restart from the stalled sites, a function that is not shared by other FA proteins and BRCA2. A unique finding of our study is that the physical presence of RAD51 paralogs at transiently stalled forks prevents generation of replication-associated DSBs, a separation of function from their role in DSB repair after fork collapse.

Increased fork stalling often yields unresolved replication intermediates which prevent proper chromosome segregation, resulting in increased micro-nucleation and formation of 53BP1 nuclear bodies in G1 daughter cells (57,58). Strikingly, we find elevated 53BP1-OPT domain formation in the absence of RAD51C, XRCC2 and XRCC3 with an anticipated increase in micro-nucleation. Notably, we show that RAD51C and XRCC3 defective cells are sensitive to both acute and chronic HU stress and exhibit increased chromosomal aberrations, highlighting their role in resolving replication problems before and after the formation of DSBs.

RAD51 is known to form foci in S-phase in the absence of DNA damage (65). In agreement with this, RAD51 paralogs were enriched spontaneously onto the S-phase chromatin, supporting their role in replication related transactions. Contrary to our expectation, RAD51 and RAD51 paralogs exhibited very weak interaction with nascent DNA during active unperturbed conditions, and RAD51C, XRCC2 and XRCC3 were found to be dispensable for regulating replication rate. Interestingly, replication fork stalling induced by various agents resulted in rapid loading of RAD51 and paralogs at nascent strands (Figure 8E). Our analyses with nascent strands after HU treatment show for the first time that three different complexes of RAD51 paralogs are found at replication pause site (Figure 8E). Consistent with previous reports (66,67), BC and DX2 but not CX3 complexes regulate RAD51 loading onto the nascent strands. This was further supported by the observation that spontaneously arising RAD51 foci were abrogated in RAD51C and XRCC2 mutant cells. These results imply that XRCC3 might play a crucial role in the late responses after fork stalling. Furthermore, specific accumulation of RAD51 paralogs onto S-phase chromatin and at CFSs in response to replication stress further supports their crucial role in resolving the replication problems. Notably, this enrichment of RAD51 paralogs was dependent on CDK activity. However, whether targeting of RAD51 paralogs to stalled replication sites depends on CDK mediated phosphorylation or due to an indirect effect of MRN complex remains unknown. Previously, we reported that RAD51C and XRCC3 focus formation is dependent on MRN complex (25). It is probable that CDK mediated limited processing of replication forks through MRN provides appropriate binding site for RAD51 paralogs. Moreover, XRCC2 possesses putative ATM/ATR target SQ motif (87), and the fact that XRCC3 S225 is phosphorylated by ATR in response to fork collapse (25,87), it will be worth understanding the role of ATR signaling in the loading/stabilization of distinct complexes of RAD51 paralogs at the replication site.

Our data show that replication forks are degraded after treatment with HU in RAD51 paralog–deficient cells by the action of MRE11 nuclease. Moreover, depletion of XRCC2 and RAD51C but not XRCC3 from BRCA2 or FANCD2 defective cells led to further fork degradation. These data points toward redundant roles of FANCD2/BRCA2 and RAD51C/XRCC2 of BCDX2 for replication fork protection. These results clearly provide extended roles of RAD51 paralogs along with RAD51 and FA-BRCA proteins in the replication fork repair pathway. RAD51 paralogs also promote restart of stalled replication forks. Interestingly, unlike RAD51C and XRCC3, XRCC2 was dispensable for replication restart. Consistent with previous reports, replication recovery was unaffected in BRCA2 deficient cells (14,19) but depletion of XRCC3 in either irs1 or V-C8 cells led to complete loss of replication restart, suggesting that replication recovery in BRCA2 or XRCC2 deficiency is taken care by CX3 complex. Recently, RAD51 and XRCC3 were shown to promote replication restart by some unknown mechanism independent of their role in HR (44). Interestingly, the only recognizable motif RAD51 paralogs possess are Walker motifs (73,77). Our analysis with Walker A mutants of RAD51C, XRCC2 and XRCC3 revealed that ATP binding by RAD51C and XRCC3 was essential and sufficient for fork stability. However, RAD51C and XRCC3 mediated ATP hydrolysis was found to be critical for restart. ATP hydrolysis by RAD51C and XRCC3 disengaged RAD51 and other paralogs from the site of stalled replication to facilitate continuous DNA synthesis (Figure 8E). A recent study shows that WRN protects the collapsed fork but is dispensable when there is an acute replication stress (88). Indeed, the recruitment of WRN at replication forks in KR XRCC3 and RAD51C cells indicates toward the collapse of forks in the absence of ATP hydrolysis. For the first time to our knowledge, we provide the molecular function of ATP hydrolysis by RAD51 paralogs. This mechanism is temporally regulated, as entrapped RAD51 and RAD51 paralogs at the stalled replication sites leads to collapsing of replication fork which is not due to fork degradation (Figure 8E). These results corroborate the recent findings of *Caenorhabditis elegans* RAD51 paralogs in modulating the RAD51 nucleoprotein filament (89).

Stalled forks are remodeled and perhaps undergo restart by different mechanisms (48). Recently, RAD51 is also implicated in fork remodeling to promote replication restart (73,90,91). Here, we provide molecular clue that fork remodeling is probably coupled with the timely disengagement of RAD51 and RAD51 paralogs, which is required to suppress excessive fork remodeling and facilitate re-initiation of replication (Figure 8E). This was further supported by the observation that depletion of FANCM translocase abrogated the enrichment of structure specific nucleases MUS81 and SLX4 at nascent DNA and CFSs in paralog deficient cells. We suggest that when either the leading or lagging strand synthesis is blocked, RAD51 paralogs with FA-BRCA proteins stabilize the fork and helicases/translocases may remodel the replication fork to re‐couple leading and lagging strand DNA synthesis (48,73,92). The concerted action of DNA synthesis combined with fork remodeling might be sensed by CX3, and thereby provide a signal for ATP hydrolysis and subsequent disengagement of RAD51 and RAD51 paralogs (Figure 8E).

RAD51C mutation leads to FA-like disorder, and are associated with inherited breast cancer (20,21). Previous analysis of RAD51C pathological mutants showed a more prominent role of RAD51C in DSB repair which might contribute to the suppression of FA and subset of breast and ovarian cancer (26,78,79). Our analysis of RAD51C pathological mutants revealed that fork protection and restart were defective in those cells. Given the importance of replicative stress in cancer development and HR defect in breast cancer pathogenesis (6), both defects seem to contribute for tumorigenesis in paralog defective tissue.

In conclusion, we have identified a novel role of RAD51 paralogs in filling the gaps of fork stability and replication restart with minimal fork processing. Distinct complexes of RAD51 paralogs prevent nucleolytic degradation of stalled forks and promote the restart of halted replication to maintain genomic integrity; failure to which leads to under-replicated genomic regions in paralogs deficient cells, and consequently tumorigenesis.

## Supplementary Material

SUPPLEMENTARY DATA

## References

[1] Yates L.R., Campbell P.J. (2012). Evolution of the cancer genome. Nat. Rev. Genet..

[2] Bakhoum S.F., Compton D.A. (2012). Chromosomal instability and cancer: a complex relationship with therapeutic potential. J. Clin. Investig..

[3] Bakhoum S.F., Compton D.A. (2009). Cancer: CINful centrosomes. Curr. Biol..

[4] Heng H.H., Bremer S.W., Stevens J.B., Horne S.D., Liu G., Abdallah B.Y., Ye K.J., Ye C.J. (2013). Chromosomal instability (CIN): what it is and why it is crucial to cancer evolution. Cancer Metastasis Rev..

[5] Burrell R.A., McGranahan N., Bartek J., Swanton C. (2013). The causes and consequences of genetic heterogeneity in cancer evolution. Nature.

[6] Burrell R.A., McClelland S.E., Endesfelder D., Groth P., Weller M.C., Shaikh N., Domingo E., Kanu N., Dewhurst S.M., Gronroos E. (2013). Replication stress links structural and numerical cancer chromosomal instability. Nature.

[7] McGranahan N., Burrell R.A., Endesfelder D., Novelli M.R., Swanton C. (2012). Cancer chromosomal instability: therapeutic and diagnostic challenges. EMBO Rep..

[8] Aguilera A., Garcia-Muse T. (2013). Causes of genome instability. Annu. Rev. Genet..

[9] Halazonetis T.D., Gorgoulis V.G., Bartek J. (2008). An oncogene-induced DNA damage model for cancer development. Science.

[10] Lecona E., Fernandez-Capetillo O. (2014). Replication stress and cancer: it takes two to tango. Exp. Cell Res..

[11] Poli J., Tsaponina O., Crabbe L., Keszthelyi A., Pantesco V., Chabes A., Lengronne A., Pasero P. (2012). dNTP pools determine fork progression and origin usage under replication stress. EMBO J..

[12] Woo Y.H., Li W.H. (2012). DNA replication timing and selection shape the landscape of nucleotide variation in cancer genomes. Nat. Commun..

[13] Hashimoto Y., Chaudhuri A.R., Lopes M., Costanzo V. (2010). Rad51 protects nascent DNA from Mre11-dependent degradation and promotes continuous DNA synthesis. Nat. Struct. Mol. Biol..

[14] Schlacher K., Christ N., Siaud N., Egashira A., Wu H., Jasin M. (2011). Double-strand break repair-independent role for BRCA2 in blocking stalled replication fork degradation by MRE11. Cell.

[15] Schlacher K., Wu H., Jasin M. (2012). A Distinct Replication Fork Protection Pathway Connects Fanconi Anemia Tumor Suppressors to RAD51-BRCA1/2. Cancer Cell.

[16] Lossaint G., Larroque M., Ribeyre C., Bec N., Larroque C., Decaillet C., Gari K., Constantinou A. (2013). FANCD2 binds MCM proteins and controls replisome function upon activation of s phase checkpoint signaling. Mol. Cell.

[17] Zeman M.K., Cimprich K.A. (2014). Causes and consequences of replication stress. Nat. Cell Biol..

[18] Langevin F., Crossan G.P., Rosado I.V., Arends M.J., Patel K.J. (2011). Fancd2 counteracts the toxic effects of naturally produced aldehydes in mice. Nature.

[19] Ying S., Hamdy F.C., Helleday T. (2012). Mre11-dependent degradation of stalled DNA replication forks is prevented by BRCA2 and PARP1. Cancer Res..

[20] Suwaki N., Klare K., Tarsounas M. (2011). RAD51 paralogs: roles in DNA damage signalling, recombinational repair and tumorigenesis. Semin. Cell. Dev. Biol..

[21] Somyajit K., Subramanya S., Nagaraju G. (2010). RAD51C: a novel cancer susceptibility gene is linked to Fanconi anemia and breast cancer. Carcinogenesis.

[22] Thacker J. (2005). The RAD51 gene family, genetic instability and cancer. Cancer Lett..

[23] Nagaraju G., Hartlerode A., Kwok A., Chandramouly G., Scully R. (2009). XRCC2 and XRCC3 regulate the balance between short- and long-tract gene conversions between sister chromatids. Mol. Cell. Biol..

[24] Nagaraju G., Odate S., Xie A., Scully R. (2006). Differential regulation of short- and long-tract gene conversion between sister chromatids by Rad51C. Mol. Cell. Biol..

[25] Somyajit K., Basavaraju S., Scully R., Nagaraju G. (2013). ATM- and ATR-mediated phosphorylation of XRCC3 regulates DNA double-strand break-induced checkpoint activation and repair. Mol. Cell. Biol..

[26] Somyajit K., Subramanya S., Nagaraju G. (2012). Distinct roles of FANCO/RAD51C protein in DNA damage signaling and repair: implications for Fanconi anemia and breast cancer susceptibility. J. Biol. Chem..

[27] Pittman D.L., Schimenti J.C. (2000). Midgestation lethality in mice deficient for the RecA-related gene, Rad51d/Rad51l3. Genesis.

[28] Shu Z., Smith S., Wang L., Rice M.C., Kmiec E.B. (1999). Disruption of muREC2/RAD51L1 in mice results in early embryonic lethality which can Be partially rescued in a p53(-/-) background. Mol. Cell. Biol..

[29] Deans B., Griffin C.S., Maconochie M., Thacker J. (2000). Xrcc2 is required for genetic stability, embryonic neurogenesis and viability in mice. EMBO J..

[30] Kuznetsov S.G., Haines D.C., Martin B.K., Sharan S.K. (2009). Loss of Rad51c leads to embryonic lethality and modulation of Trp53-dependent tumorigenesis in mice. Cancer Res..

[31] Orr N., Lemnrau A., Cooke R., Fletcher O., Tomczyk K., Jones M., Johnson N., Lord C.J., Mitsopoulos C., Zvelebil M. (2012). Genome-wide association study identifies a common variant in RAD51B associated with male breast cancer risk. Nat. Genet..

[32] Meindl A., Hellebrand H., Wiek C., Erven V., Wappenschmidt B., Niederacher D., Freund M., Lichtner P., Hartmann L., Schaal H. (2010). Germline mutations in breast and ovarian cancer pedigrees establish RAD51C as a human cancer susceptibility gene. Nat. Genet..

[33] Osorio A., Endt D., Fernandez F., Eirich K., de la Hoya M., Schmutzler R., Caldes T., Meindl A., Schindler D., Benitez J. (2012). Predominance of pathogenic missense variants in the RAD51C gene occurring in breast and ovarian cancer families. Hum. Mol. Genet..

[34] Loveday C., Turnbull C., Ramsay E., Hughes D., Ruark E., Frankum J.R., Bowden G., Kalmyrzaev B., Warren-Perry M., Snape K. (2011). Germline mutations in RAD51D confer susceptibility to ovarian cancer. Nat. Genet..

[35] Park D.J., Lesueur F., Nguyen-Dumont T., Pertesi M., Odefrey F., Hammet F., Neuhausen S.L., John E.M., Andrulis I.L., Terry M.B. (2012). Rare mutations in XRCC2 increase the risk of breast cancer. Am. J. Hum. Genet..

[36] Golmard L., Caux-Moncoutier V., Davy G., Al Ageeli E., Poirot B., Tirapo C., Michaux D., Barbaroux C., d'Enghien C.D., Nicolas A. (2013). Germline mutation in the RAD51B gene confers predisposition to breast cancer. BMC Cancer.

[37] Shamseldin H.E., Elfaki M., Alkuraya F.S. (2012). Exome sequencing reveals a novel Fanconi group defined by XRCC2 mutation. J. Med. Genet..

[38] Vaz F., Hanenberg H., Schuster B., Barker K., Wiek C., Erven V., Neveling K., Endt D., Kesterton I., Autore F. (2010). Mutation of the RAD51C gene in a Fanconi anemia-like disorder. Nat. Genet..

[39] Badie S., Liao C., Thanasoula M., Barber P., Hill M.A., Tarsounas M. (2009). RAD51C facilitates checkpoint signaling by promoting CHK2 phosphorylation. J. Cell Biol..

[40] Rodrigue A., Lafrance M., Gauthier M.C., McDonald D., Hendzel M., West S.C., Jasin M., Masson J.Y. (2006). Interplay between human DNA repair proteins at a unique double-strand break in vivo. EMBO J..

[41] Compton S.A., Ozgur S., Griffith J.D. (2010). Ring-shaped Rad51 paralog protein complexes bind Holliday junctions and replication forks as visualized by electron microscopy. J. Biol. Chem..

[42] Liu N., Lim C.S. (2005). Differential roles of XRCC2 in homologous recombinational repair of stalled replication forks. J. Cell. Biochem..

[43] Henry-Mowatt J., Jackson D., Masson J.Y., Johnson P.A., Clements P.M., Benson F.E., Thompson L.H., Takeda S., West S.C., Caldecott K.W. (2003). XRCC3 and Rad51 modulate replication fork progression on damaged vertebrate chromosomes. Mol. Cell.

[44] Petermann E., Orta M.L., Issaeva N., Schultz N., Helleday T. (2010). Hydroxyurea-stalled replication forks become progressively inactivated and require two different RAD51-mediated pathways for restart and repair. Mol. Cell.

[45] Rodrigue A., Coulombe Y., Jacquet K., Gagne J.P., Roques C., Gobeil S., Poirier G., Masson J.Y. (2013). The RAD51 paralogs ensure cellular protection against mitotic defects and aneuploidy. J. Cell Sci..

[46] Daboussi F., Courbet S., Benhamou S., Kannouche P., Zdzienicka M.Z., Debatisse M., Lopez B.S. (2008). A homologous recombination defect affects replication-fork progression in mammalian cells. J. Cell Sci..

[47] Hashimoto Y., Ray Chaudhuri A., Lopes M., Costanzo V. (2010). Rad51 protects nascent DNA from Mre11-dependent degradation and promotes continuous DNA synthesis. Nat. Struct. Mol. Biol..

[48] Petermann E., Helleday T. (2010). Pathways of mammalian replication fork restart. Nat. Rev. Mol. Cell. Biol..

[49] Lundin C., Schultz N., Arnaudeau C., Mohindra A., Hansen L.T., Helleday T. (2003). RAD51 is involved in repair of damage associated with DNA replication in mammalian cells. J. Mol. Biol..

[50] Xue Y., Li Y., Guo R., Ling C., Wang W. (2008). FANCM of the Fanconi anemia core complex is required for both monoubiquitination and DNA repair. Hum. Mol. Genet..

[51] Toledo L.I., Altmeyer M., Rask M.B., Lukas C., Larsen D.H., Povlsen L.K., Bekker-Jensen S., Mailand N., Bartek J., Lukas J. (2013). ATR Prohibits Replication Catastrophe by Preventing Global Exhaustion of RPA. Cell.

[52] Ragland R.L., Patel S., Rivard R.S., Smith K., Peters A.A., Bielinsky A.K., Brown E.J. (2013). RNF4 and PLK1 are required for replication fork collapse in ATR-deficient cells. Genes Dev..

[53] Hanada K., Budzowska M., Davies S.L., van Drunen E., Onizawa H., Beverloo H.B., Maas A., Essers J., Hickson I.D., Kanaar R. (2007). The structure-specific endonuclease Mus81 contributes to replication restart by generating double-strand DNA breaks. Nat. Struct. Mol. Biol..

[54] Urbin S.S., Elvers I., Hinz J.M., Helleday T., Thompson L.H. (2012). Uncoupling of RAD51 focus formation and cell survival after replication fork stalling in RAD51D null CHO cells. Environ. Mol. Mutagen..

[55] Godthelp B.C., Wiegant W.W., van Duijn-Goedhart A., Scharer O.D., van Buul P.P., Kanaar R., Zdzienicka M.Z. (2002). Mammalian Rad51C contributes to DNA cross-link resistance, sister chromatid cohesion and genomic stability. Nucleic Acids Res..

[56] Elvers I., Johansson F., Groth P., Erixon K., Helleday T. (2011). UV stalled replication forks restart by re-priming in human fibroblasts. Nucleic Acids Res..

[57] Lukas C., Savic V., Bekker-Jensen S., Doil C., Neumann B., Pedersen R.S., Grofte M., Chan K.L., Hickson I.D., Bartek J. (2011). 53BP1 nuclear bodies form around DNA lesions generated by mitotic transmission of chromosomes under replication stress. Nat. Cell Biol..

[58] Harrigan J.A., Belotserkovskaya R., Coates J., Dimitrova D.S., Polo S.E., Bradshaw C.R., Fraser P., Jackson S.P. (2011). Replication stress induces 53BP1-containing OPT domains in G1 cells. J. Cell Biol..

[59] Arlt M.F., Casper A.M., Glover T.W. (2003). Common fragile sites. Cytogenet Genome Res..

[60] Casper A.M., Nghiem P., Arlt M.F., Glover T.W. (2002). ATR regulates fragile site stability. Cell.

[61] Glover T.W. (1998). Instability at chromosomal fragile sites. Recent Results Cancer Res..

[62] Glover T.W., Berger C., Coyle J., Echo B. (1984). DNA polymerase alpha inhibition by aphidicolin induces gaps and breaks at common fragile sites in human chromosomes. Hum. Genet..

[63] Glover T.W., Coyle-Morris J., Morgan R. (1986). Fragile sites: overview, occurrence in acute nonlymphocytic leukemia and effects of caffeine on expression. Cancer Genet. Cytogenet..

[64] Lu X., Parvathaneni S., Hara T., Lal A., Sharma S. (2013). Replication stress induces specific enrichment of RECQ1 at common fragile sites FRA3B and FRA16D. Mol. Cancer.

[65] Tarsounas M., Davies D., West S.C. (2003). BRCA2-dependent and independent formation of RAD51 nuclear foci. Oncogene.

[66] Masson J.Y., Tarsounas M.C., Stasiak A.Z., Stasiak A., Shah R., McIlwraith M.J., Benson F.E., West S.C. (2001). Identification and purification of two distinct complexes containing the five RAD51 paralogs. Genes Dev..

[67] Chun J., Buechelmaier E.S., Powell S.N. (2013). Rad51 Paralog Complexes BCDX2 and CX3 Act at Different Stages in the BRCA1-BRCA2-Dependent Homologous Recombination Pathway. Mol. Cell. Biol..

[68] Sirbu B.M., McDonald W.H., Dungrawala H., Badu-Nkansah A., Kavanaugh G.M., Chen Y., Tabb D.L., Cortez D. (2013). Identification of proteins at active, stalled, and collapsed replication forks using isolation of proteins on nascent DNA (iPOND) coupled with mass spectrometry. J. Biol. Chem..

[69] Altmeyer M., Lukas J. (2013). Guarding against collateral damage during chromatin transactions. Cell.

[70] Altmeyer M., Lukas J. (2013). To spread or not to spread–chromatin modifications in response to DNA damage. Curr. Opin. Genet. Dev..

[71] Jensen R.B., Ozes A., Kim T., Estep A., Kowalczykowski S.C. (2013). BRCA2 is epistatic to the RAD51 paralogs in response to DNA damage. DNA Repair (Amst.).

[72] Qing Y., Yamazoe M., Hirota K., Dejsuphong D., Sakai W., Yamamoto K.N., Bishop D.K., Wu X., Takeda S. (2011). The epistatic relationship between BRCA2 and the other RAD51 mediators in homologous recombination. PLoS Genet..

[73] Neelsen K.J., Lopes M. (2015). Replication fork reversal in eukaryotes: from dead end to dynamic response. Nat. Rev. Mol. Cell Biol..

[74] Couch F.B., Bansbach C.E., Driscoll R., Luzwick J.W., Glick G.G., Betous R., Carroll C.M., Jung S.Y., Qin J., Cimprich K.A. (2013). ATR phosphorylates SMARCAL1 to prevent replication fork collapse. Genes Dev..

[75] Schwab R.A., Blackford A.N., Niedzwiedz W. (2010). ATR activation and replication fork restart are defective in FANCM-deficient cells. EMBO J..

[76] Luke-Glaser S., Luke B., Grossi S., Constantinou A. (2010). FANCM regulates DNA chain elongation and is stabilized by S-phase checkpoint signalling. EMBO J..

[77] Miller K.A., Sawicka D., Barsky D., Albala J.S. (2004). Domain mapping of the Rad51 paralog protein complexes. Nucleic Acids Res..

[78] Somyajit K., Mishra A., Jameei A., Nagaraju G. (2015). Enhanced non-homologous end joining contributes toward synthetic lethality of pathological RAD51C mutants with poly (ADP-ribose) polymerase. Carcinogenesis.

[79] Park J.Y., Singh T.R., Nassar N., Zhang F., Freund M., Hanenberg H., Meetei A.R., Andreassen P.R. (2014). Breast cancer-associated missense mutants of the PALB2 WD40 domain, which directly binds RAD51C, RAD51 and BRCA2, disrupt DNA repair. Oncogene.

[80] Neelsen K.J., Zanini I.M., Herrador R., Lopes M. (2013). Oncogenes induce genotoxic stress by mitotic processing of unusual replication intermediates. J. Cell Biol..

[81] Crasta K., Ganem N.J., Dagher R., Lantermann A.B., Ivanova E.V., Pan Y., Nezi L., Protopopov A., Chowdhury D., Pellman D. (2012). DNA breaks and chromosome pulverization from errors in mitosis. Nature.

[82] Zhu W., Abbas T., Dutta A. (2005). DNA replication and genomic instability. Adv. Exp. Med. Biol..

[83] Durkin S.G., Glover T.W. (2007). Chromosome fragile sites. Annu. Rev. Genet..

[84] Branzei D., Foiani M. (2010). Maintaining genome stability at the replication fork. Nat. Rev. Mol. Cell. Biol..

[85] Osborn A.J., Elledge S.J., Zou L. (2002). Checking on the fork: the DNA-replication stress-response pathway. Trends Cell Biol..

[86] Roy R., Chun J., Powell S.N. (2012). BRCA1 and BRCA2: different roles in a common pathway of genome protection. Nat. Rev. Cancer.

[87] Matsuoka S., Ballif B.A., Smogorzewska A., McDonald E.R. 3rd, Hurov K.E., Luo J., Bakalarski C.E., Zhao Z., Solimini N., Lerenthal Y. (2007). ATM and ATR substrate analysis reveals extensive protein networks responsive to DNA damage. Science.

[88] Su F., Mukherjee S., Yang Y., Mori E., Bhattacharya S., Kobayashi J., Yannone S.M., Chen D.J., Asaithamby A. (2014). Nonenzymatic role for WRN in preserving nascent DNA strands after replication stress. Cell Rep..

[89] Taylor M.R., Spirek M., Chaurasiya K.R., Ward J.D., Carzaniga R., Yu X., Egelman E.H., Collinson L.M., Rueda D., Krejci L. (2015). Rad51 Paralogs Remodel Pre-synaptic Rad51 Filaments to Stimulate Homologous Recombination. Cell.

[90] Thangavel S., Berti M., Levikova M., Pinto C., Gomathinayagam S., Vujanovic M., Zellweger R., Moore H., Lee E.H., Hendrickson E.A. (2015). DNA2 drives processing and restart of reversed replication forks in human cells. J. Cell Biol..

[91] Zellweger R., Dalcher D., Mutreja K., Berti M., Schmid J.A., Herrador R., Vindigni A., Lopes M. (2015). Rad51-mediated replication fork reversal is a global response to genotoxic treatments in human cells. J. Cell Biol..

[92] Fugger K., Mistrik M., Neelsen K.J., Yao Q., Zellweger R., Kousholt A.N., Haahr P., Chu W.K., Bartek J., Lopes M. (2015). FBH1 Catalyzes Regression of Stalled Replication Forks. Cell Rep..

