# Mammalian RAD51 paralogs protect nascent DNA at stalled forks and mediate replication restart

**DOI:** 10.1093/nar/gkaa279

**Published:** 2020-04-17

**Authors:** Kumar Somyajit, Sneha Saxena, Sharath Babu, Anup Mishra, Ganesh Nagaraju

**Affiliations:** Department of Biochemistry, Indian Institute of Science, Bangalore-560012, India

*Nucleic Acids Research*, 2015, 43(20): 9835–9855, https://doi.org/10.1093/nar/gkv880

In Figure 2A, the representative images for XRCC2 shRNA and XRCC3 shRNA are identical. The representative image for 53BP1 NBs after XRCC3 knockdown was erroneously duplicated during figure assembly. The error has now been rectified with the correct representative image, a new figure is provided below. This error does not alter the results and conclusions of this article.

**Figure 2.**
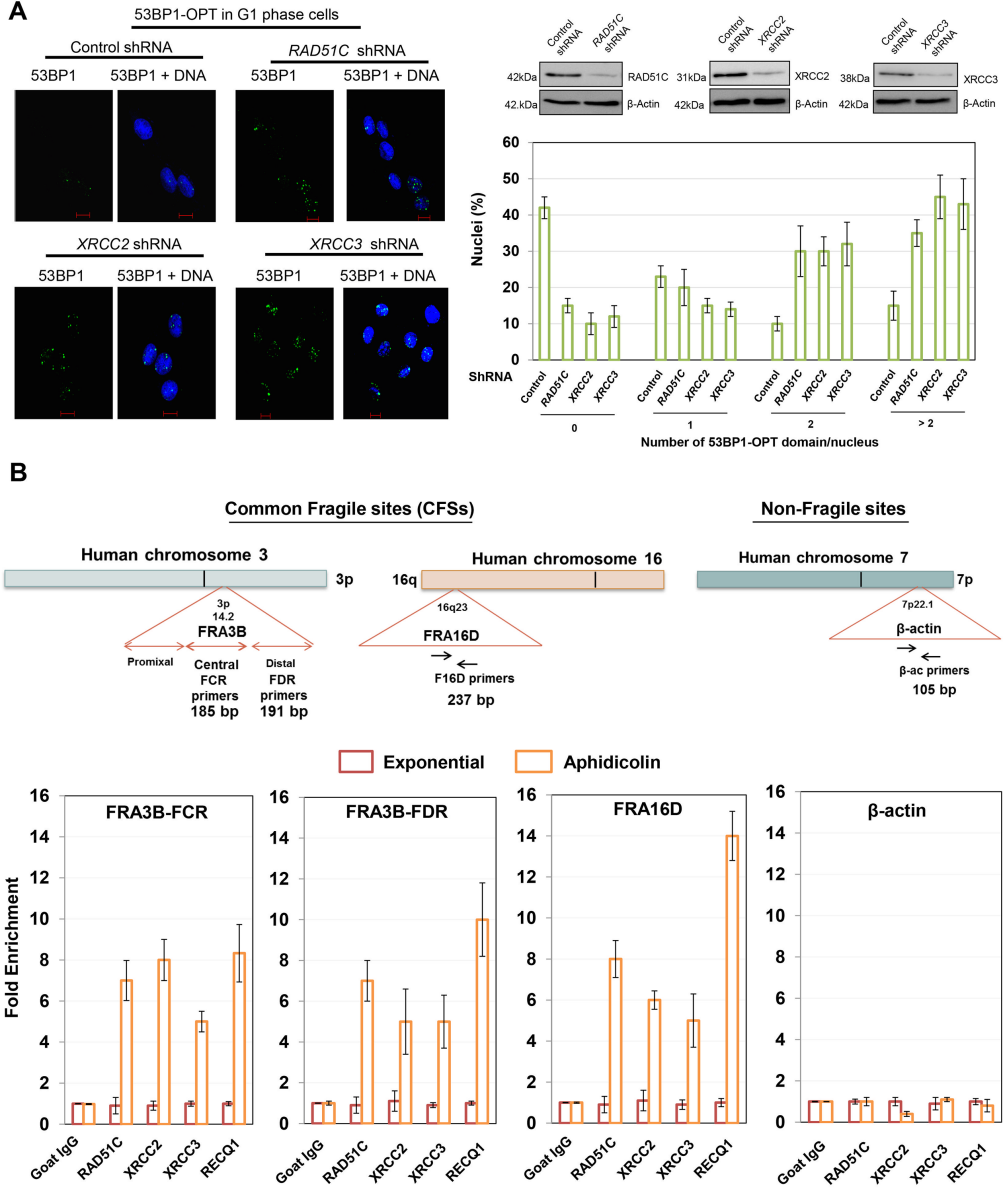
RAD51 paralogs localize to Common Fragile Sites (CFSs) upon replicative stress and suppress replication associated lesions. (**A**) Representative images and quantification of G1 phase synchronized U2OS control and RAD51C, XRCC2 and XRCC3 depleted cells, fixed and stained with 53BP1 antibody to visualize OPT domains (53BP1-green andDNA-blue). At least 100 G1 cells were counted for each experiment. Blots for depletion of RAD51C, XRCC2 and XRCC3 have been shown. (**B**) Genomic organization of the FRA3B, FRA16D and β-Actin (ACTB) region. Primer sets of distal (FDR) and central (FCR) region within the FRA3B locus, FRA16D locus and β-Actin locus are indicated. Quantification of cross-linked FRA3B-FCR, FRA3BFDR, FRA16D and β-Actin loci chromatin immunoprecipitated from HeLa cells using indicated antibodies. RECQ1 antibody was used as a positive control for FRA3B and FRA16D enrichment. Fold enrichment over goat IgG was determined and is shown for each primer pair for the ChIP. Results are expressed as mean ± SD for at least three independent experiments.

