# Supplementary material for: Mammalian RAD51 paralogs protect nascent DNA at stalled forks and mediate replication restart
Source: Nucleic Acids Res. 2015 Sep 9;43(20):9835–55. doi: 10.1093/nar/gkv880 (PMC4787763; doi:10.1093/nar/gkv880)
Supplement: SUPPLEMENTARY DATA [file supp_43_20_9835__index.html]

Mammalian RAD51 paralogs protect nascent DNA at stalled forks and mediate replication restart — SUPPLEMENTARY DATA 

# Mammalian RAD51 paralogs protect nascent DNA at stalled forks and mediate replication restart

## SUPPLEMENTARY DATA

- SUPPLEMENTARY DATA
