# Supplementary material for: Mammalian RAD51 paralogs protect nascent DNA at stalled forks and mediate replication restart
Source: Nucleic Acids Res. 2015 Sep 9;43(20):9835–55. doi: 10.1093/nar/gkv880 (PMC4787763; doi:10.1093/nar/gkv880)
Supplement: SUPPLEMENTARY DATA [file supp_gkv880_nar-01115-d-2015-File003.pdf]

Supplementary Figure legends

Supplementary Figure 1. RAD51 and RAD51 paralogs are enriched spontaneously onto the S-phase chromatin during DNA replication. (A) Chromatin fractionation was carried out as described in materials and methods. H2AX was used as marker for chromatin while α-tubulin and Lamin A served as marker for cytosolic and nuclear fractions, respectively. (B) U2OS cells were synchronized by serum starvation (G0/G1), thymidine-aphidicolin block (S) and nocodazole (G2/M). Whole cell extracts were prepared at indicated cell cycle stages and analysed for RAD51 and RAD51 paralogs. Asyn, asynchronous cells (C) HeLa cells were synchronized with nocodazole and at the indicated stages of the cell cycle, cells were harvested and chromatin extract was prepared and analyzed for indicated proteins. Asyn, asynchronous cells.

Supplementary Figure 2. RAD51 paralogs associate with S-phase chromatin in a CDK-dependent manner. (A) HeLa cells were synchronized in G0/G1 phase with serum starved media and cells were harvested at indicated time points and chromatin extract was prepared to analyze indicated proteins. (B) G0/G1 synchronized HeLa cells were harvested without or with addition of CDK inhibitor (last 1 h) at indicated time points and chromatin extract was prepared to analyze indicated proteins.

Supplementary Figure 3. RAD51 paralogs supress HU induced toxicity. (A) Experimental design for nascent strand pull-down of active replication forks. HeLa cells were labelled with IdU for indicated times. Cells were cross-linked, and the chromatin fraction was isolated (input) and subjected to IP using anti-IdU antibody (Idu-IP) or only beads (Beads control). Fractions were probed for indicated proteins with H3 as loading control. (B) Replication fork velocity as a measure of CldU tract length in RAD51 paralogs deficient cells compared to respective parental cells. A minimum of 100 tracts were measured per

experiment. Mean values for three independent experiments are shown±SD. (C) Cellular sensitivity of RAD51C, XRCC2 and XRCC3 deficient cells to continuous exposure of HU, CPT and APH as measured by colony formation assay. Results shown are mean±SD of three independent experiments.

Supplementary Figure 4. RAD51 paralogs associate with CFSs following replicative stress. (A) Experimental design for pull-down of nascent DNA behind fork during template damage. HeLa cells were labelled with IdU for 30 min, followed by UV-C irradiation (25J/m^2) and a *chase* into thymidine-containing medium for the indicated times. Cells were cross-linked, and the chromatin fraction was isolated (input) and subjected to IP using anti-IdU antibody. Fractions were probed for indicated proteins. (B) Genomic organization of the FRA3B and FRA16D region. Primer sets of distal (FDR) and central (FCR) region within the FRA3B locus, and for FRA16D are indicated. Experimental design for ChIP analysis. HeLa cells were either untreated or treated with aphidicolin (0.5μM) for 24 h and then processed for ChIP followed by semi-quantitative PCR using RAD51C, XRCC2 and XRCC3-specific antibody. A representative gel of the amplified DNA immunoprecipitated with indicated antibodies. Input represents 1% of the cross-linked chromatin used for ChIP. (C) Quantification of cross-linked FRA3B-FCR and FRA3B-FDR chromatin immunoprecipitated from HU treated HeLa cells using indicated antibodies. Fold enrichment over goat IgG was determined and is shown for each primer pair for the ChIP. Results are expressed as means±SD for at least three independent experiments.

Supplementary Figure 5. RAD51 paralogs are crucial for spontaneous chromatin loading of RAD51 in S-phase. (A) CDK inhibition disrupts RAD51 paralogs enrichment on the fragile sites. HeLa cells were either untreated or treated with aphidicolin (0.5 μM) for 24 h and then processed for ChIP using RAD51, RAD51C, XRCC2 and XRCC3-specific antibody after adding CDK inhibitor for last 1 h. Representative gel images are shown. Fold

enrichment over goat IgG was determined and is shown for each primer pair for the ChIP. Results are expressed as means±SD for at least three independent experiments. (B) Representative images of RAD51 foci formed spontaneously in asynchronous and S-phase V79B cells. Quantification of spontaneous RAD51 foci in S-phase in the indicated cells. At least 50 cells were counted and results show the mean of two experiments.

Supplementary Figure 6. RAD51 paralogs assemble at stalled replication forks in distinct complexes and regulate the loading of RAD51. (A) Experimental design for nascent strand pull-down of HU-mediated stalled replication forks in RAD51 paralogs deficient cells. Indicated cells were labeled with IdU for 15 min followed by addition of HU for 45 min. Cells were cross-linked, and the chromatin fraction was isolated (input) and subjected to IP using anti-IdU antibody. Fractions were probed for RAD51 and RAD51 paralogs with Histone H3 (input loading control). (B) RAD51 paralogs are critical for RAD51 recruitment to CFSs. Quantification of fold enrichment of RAD51 at FRA3B and FRA16D sites from the indicated cells. The indicated hamster cells or HeLa cells treated with control or RAD51C, XRCC2 and XRCC3 shRNA for 24 h were exposed to aphidicolin (0.5μM) for the next 24 h, and analyzed by ChIP. Data are means ±SD (n=3).

Supplementary Figure 7. Cells deficient in RAD51 paralogs undergo increased replication fork collapse following replicative stress. (A) RAD51 paralog depleted or control HeLa cells were either untreated or treated with aphidicolin (24 h) and analysed by ChIP for the γ-H2AX accumulation at the indicated CFSs (FRA16D and FRA3B) or control genomic regions (β-actin). Data are means ±SD (n=3). (B) Quantification of 53BP1 positive nuclei from 100 V79B and U2OS cells after treatment with HU for indicated time periods. Cells having 8 or more foci were considered as positive. (C) Cell cycle profile after HU and aphidicolin treatment in U20S cells.

Supplementary Figure 8. FA-BRCA pathway contributes to maintenance of stalled forks and genomic stability. (A) Cellular sensitivity to continuous exposure of PARP inhibitor (ABT-888) and 4 h of HU in BRCA2 deficient V-C8 cells. (B) Chromosomal aberrations after 4 h of HU in BRCA2 deficient V-C8 cells. (C) Measurement of IdU labelled DNA fiber in BRCA2 defective V-C8 and parental V79 cells. (D) Measurement of IdU labelled DNA fiber in I-BR3 and V79B cells treated with FANCD2 shRNA. Western blots show depletion of FANCD2 in indicated cells.

Supplementary Figure 9. RAD51 paralogs, in parallel with FA-BRCA pathway, contribute to maintenance of stalled forks and genomic stability. (A) Measurement of IdU labelled DNA fiber in BRCA2-defective V-C8 cells with or without depletion of RAD51paralogs. Bar graph represents median IdU tract lengths of 100 DNA fibers. (B) Measurement of IdU labelled DNA fiber in RAD51 paralog deficient cells with or without depletion of FANCD2. (C) Epistatic roles of RAD51 paralogs with BRCA2 in DSB repair. Cellular sensitivity to Zeocin in BRCA2 deficient V-C8 cells following depletion of RAD51 paralogs. (D) Fold enrichment of MUS81 at FRA3B-FCR locus from RAD51 paralog deficient cells. Cells were treated with aphidicolin (0.5 μM) for 24 h, and analyzed by ChIP. Data are means ±SD (n=3). (E) Fold enrichment of SLX4 at FRA3B-FCR locus from RAD51 paralog deficient cells. Cells were treated with aphidicolin (0.5 μM) for 24 h, and analyzed by ChIP. Data are means ±SD (n=3). (F) Fold enrichment of MUS81 at FRA3B-FCR locus from CL-V4B cells treated with control/FANCM shRNA or WRN inhibitor. Cells were treated with aphidicolin (0.5 μM) for 24 h, and analyzed by ChIP. Data are means ±SD (n=3).

Supplementary Figure 10. RAD51C and XRCC3 are critical for fork restart and unperturbed S-phase progression. Confocal microscopy images of representative S-phase cells during stalled or recovering replication. Cells were stained with anti-IdU (red) and anti-XRCC2 (A), anti-RAD51C (B) or anti-XRCC3 (C) antibodies. (D) Experimental protocol for

measuring S-phase progression. U2OS cells were pulse-labeled with 50 μM BrdU for 40 min, washed (W), and then treated with 4 mM HU for 2 h. HU was removed, and the S-phase population was monitored immediately after treatment (0h) and at the indicated times after removal of HU (1-24 h). FACS profiles for control and RAD51 paralogs depleted cells. (E) Spontaneously arising micro-nucleated cells and their quantification from indicated cells, as determined by propidium iodide (red) and α-tubulin (green) staining, ($n \geq 100$ asynchronous cells per experiment). Results shown are mean±SD ($n=3$). P values are obtained for paralog deficient cells in comparison with parental cells. (F) Quantification of spontaneously arising micro-nuclei in CL-V4B cells overexpressing wt or indicated RAD51C mutants.

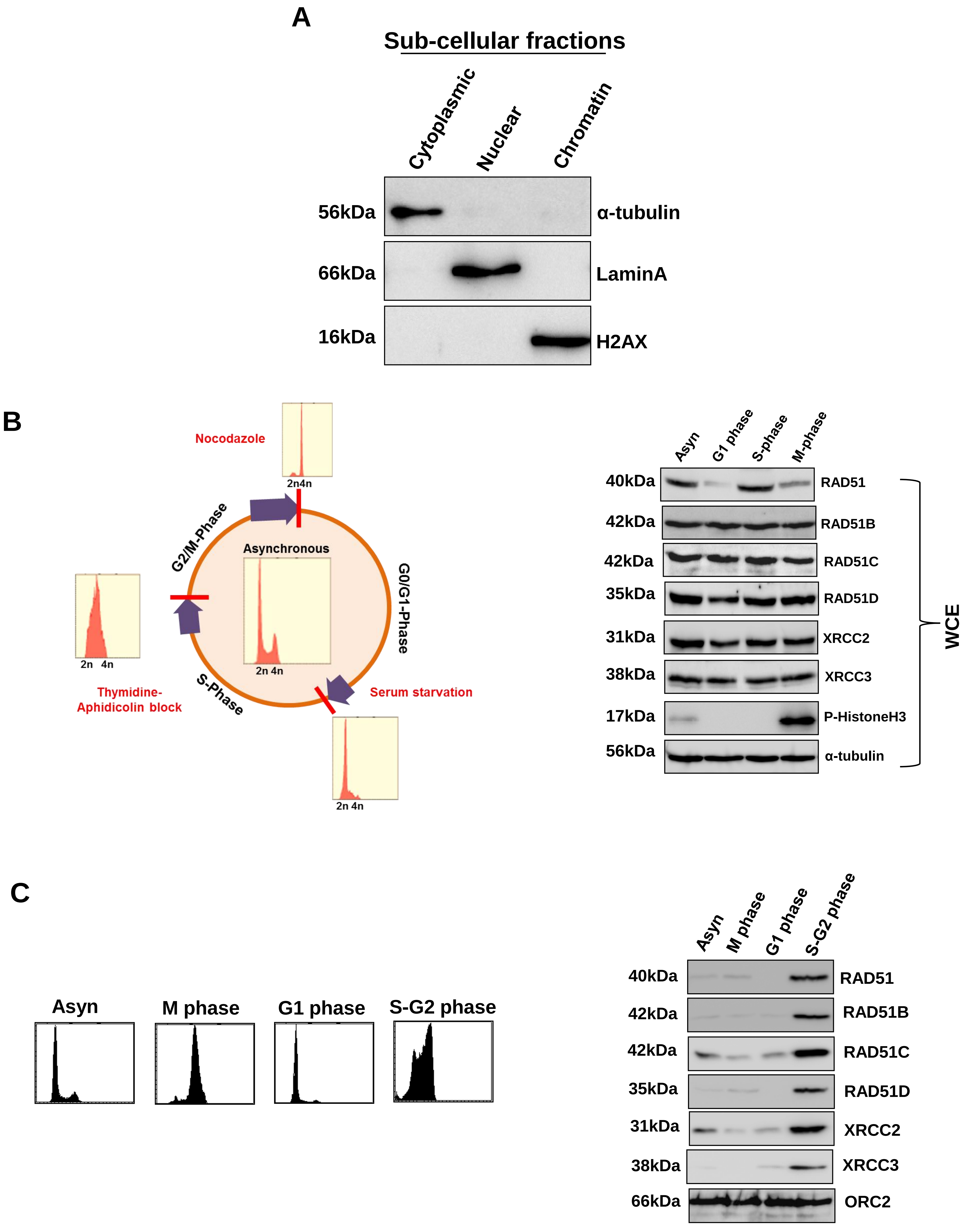
A
Sub-cellular fractions
Cytoplasmic
Nuclear
Chromatin
56kDa
α-tubulin
66kDa
LaminA
16kDa
H2AX
B
Nocodazole
2n4n
G2/M-Phase
Asynchronous
G0/G1-Phase
2n 4n
2n 4n
Thymidine-
Aphidicolin block
S-Phase
Serum starvation
2n 4n
Asyn
G1 phase
S-phase
M-phase
40kDa
RAD51
42kDa
RAD51B
42kDa
RAD51C
35kDa
RAD51D
31kDa
XRCC2
38kDa
XRCC3
17kDa
P-HistoneH3
56kDa
α-tubulin
WCE
C
Asyn
M phase
G1 phase
S-G2 phase
Asyn
M phase
G1 phase
S-G2 phase
40kDa
RAD51
42kDa
RAD51B
42kDa
RAD51C
35kDa
RAD51D
31kDa
XRCC2
38kDa
XRCC3
66kDa
ORC2

Sup Figure 1

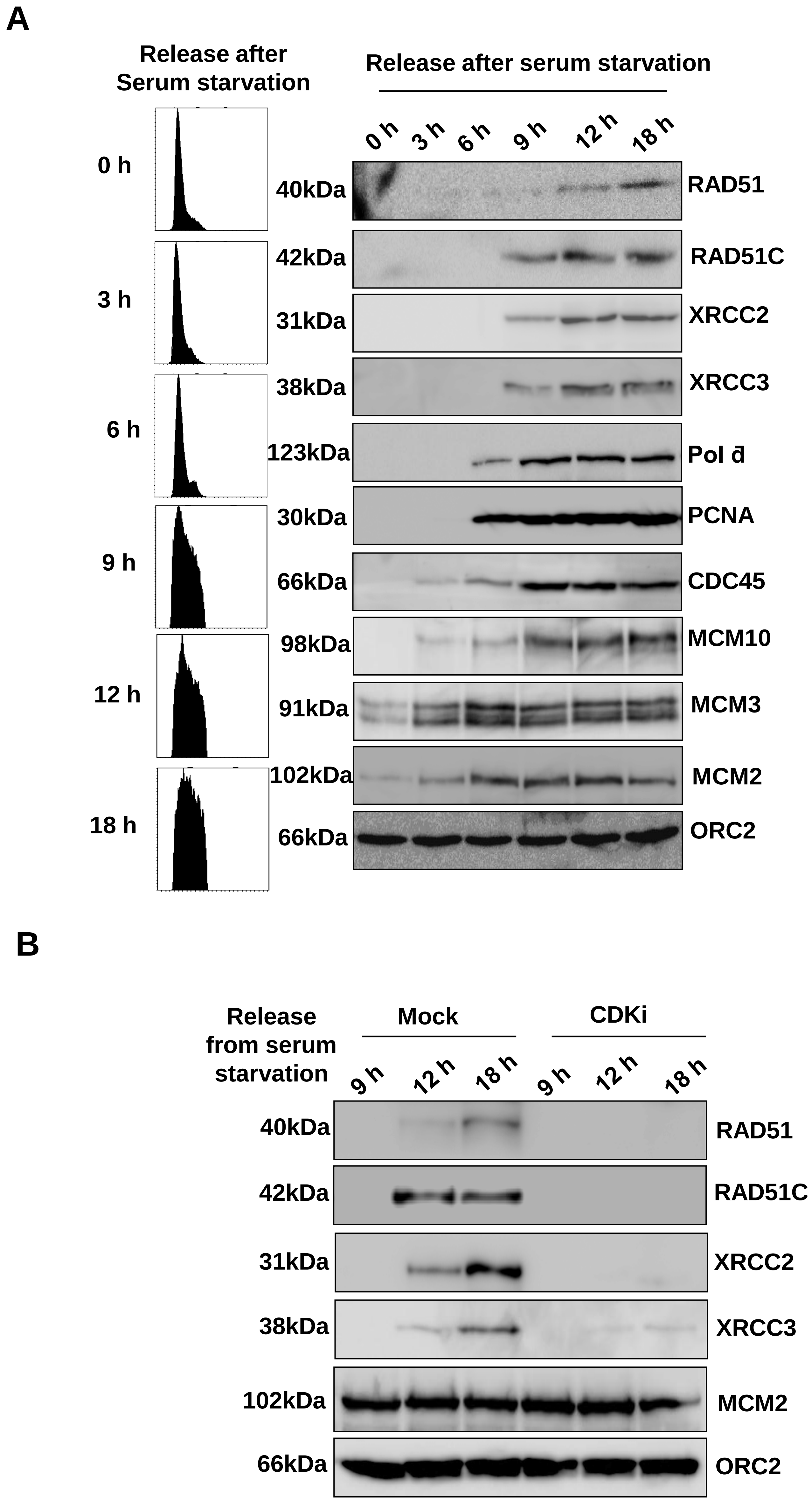
A
Release after Serum starvation
0 h
3 h
6 h
9 h
12 h
18 h
Release after serum starvation
0 h
3 h
6 h
9 h
12 h
18 h
40kDa
RAD51
42kDa
RAD51C
31kDa
XRCC2
38kDa
XRCC3
123kDa
Pol δ
30kDa
PCNA
66kDa
CDC45
98kDa
MCM10
91kDa
MCM3
102kDa
MCM2
66kDa
ORC2
B
Release from serum starvation
Mock
CDKi
9 h
12 h
18 h
9 h
12 h
18 h
40kDa
RAD51
42kDa
RAD51C
31kDa
XRCC2
38kDa
XRCC3
102kDa
MCM2
66kDa
ORC2

Sup Figure 2

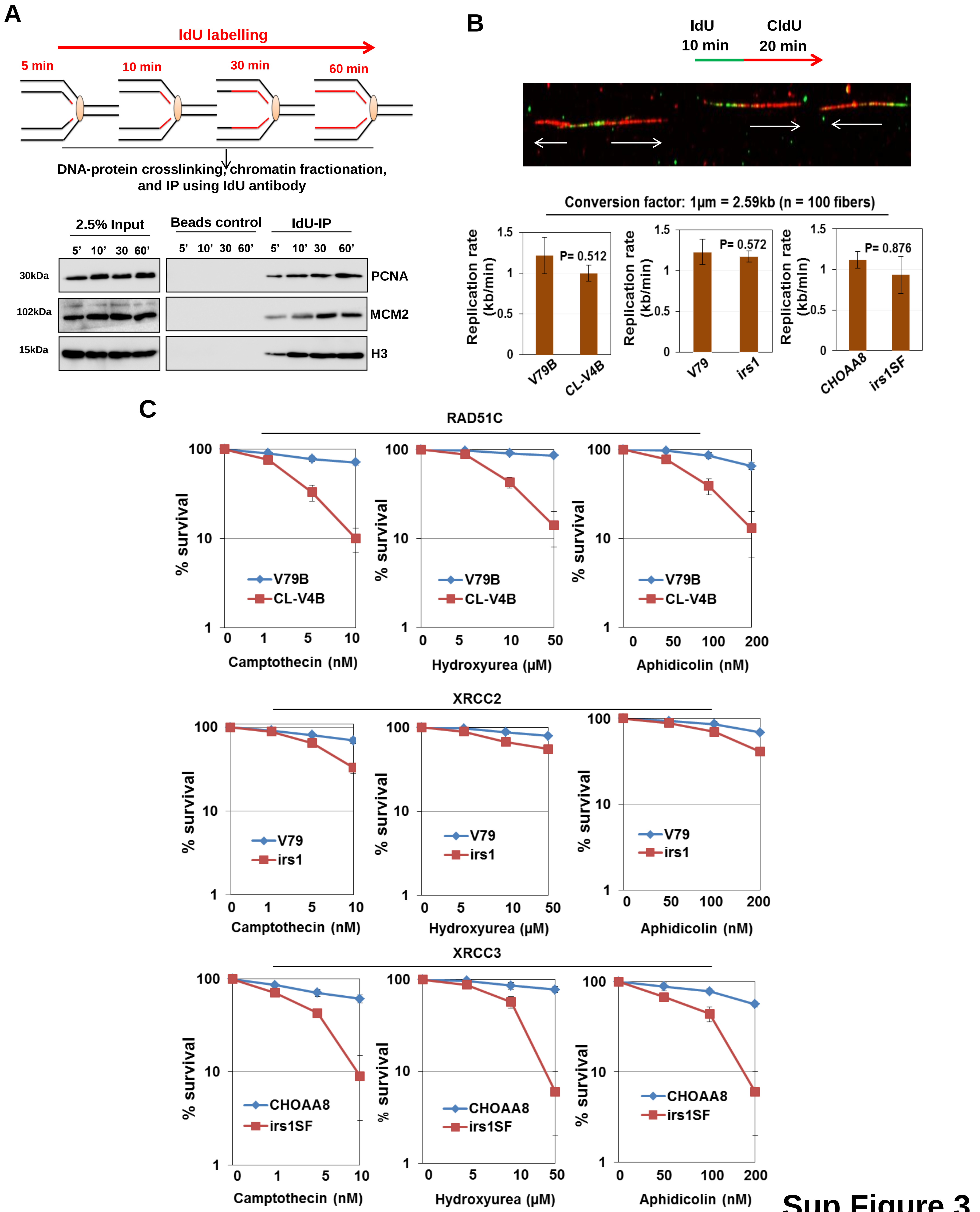

A
IdU labelling
5 min
10 min
30 min
60 min
DNA-protein crosslinking, chromatin fractionation, and IP using IdU antibody
2.5% Input
Beads control
IdU-IP
5' 10' 30 60'
30kDa
102kDa
15kDa
PCNA
MCM2
H3
B
IdU 10 min
CldU 20 min
Conversion factor: 1μm = 2.59kb (n = 100 fibers)
Replication rate (kb/min)
P= 0.512
P= 0.572
P= 0.876
V79B
CL-V4B
V79
irs1
CHOAA8
irs1SF
C
RAD51C
XRCC2
XRCC3
% survival
Camptothecin (nM)
Hydroxyurea (μM)
Aphidicolin (nM)

Sup Figure 3

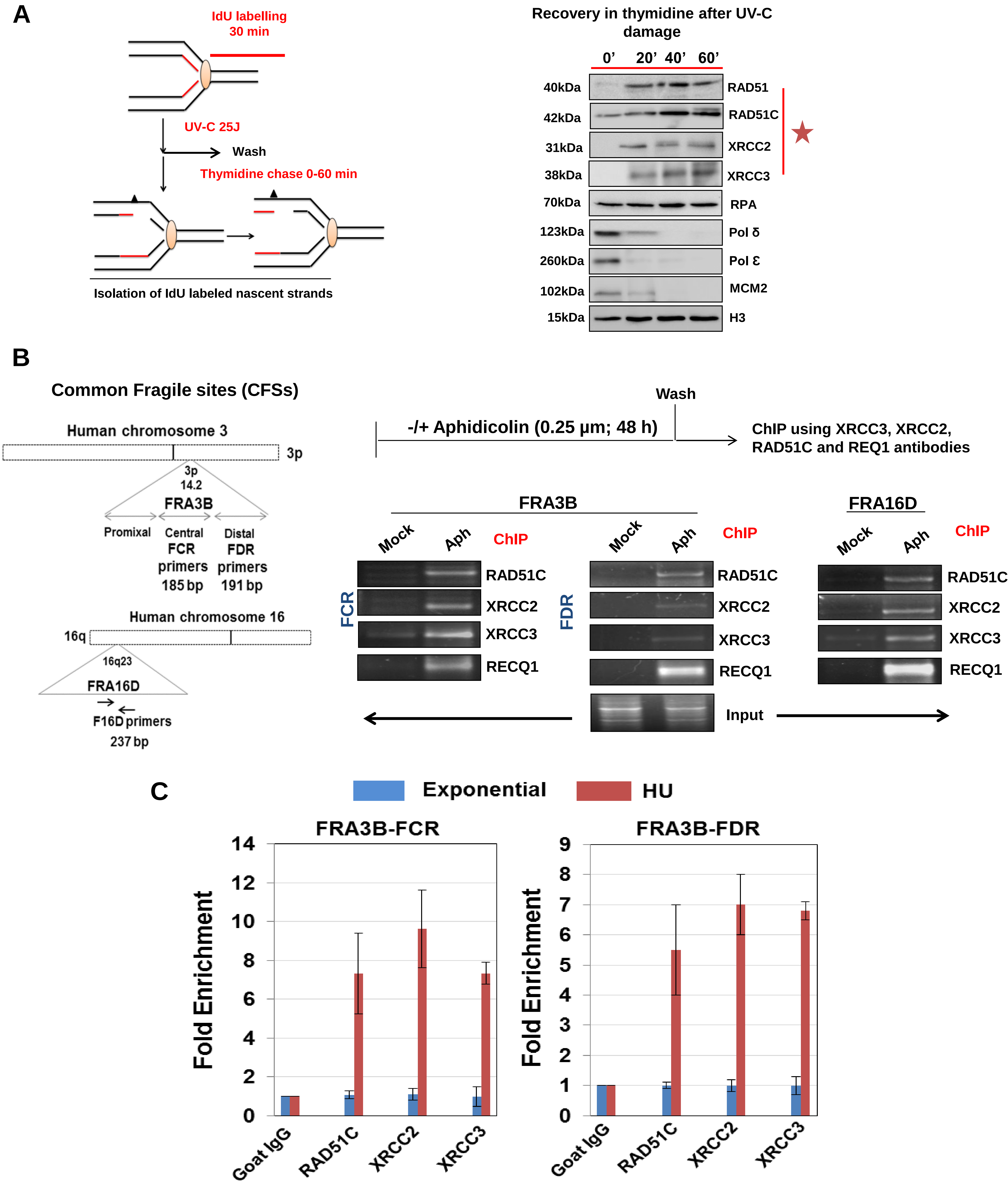
A
IdU labelling
30 min
UV-C 25J
Wash
Thymidine chase 0-60 min
Isolation of IdU labeled nascent strands
Recovery in thymidine after UV-C damage
0' 20' 40' 60'
40kDa RAD51
42kDa RAD51C
31kDa XRCC2
38kDa XRCC3
70kDa RPA
123kDa Pol δ
260kDa Pol ε
102kDa MCM2
15kDa H3
B
Common Fragile sites (CFSs)
Human chromosome 3
3p
3p 14.2
FRA3B
Promixal
Central FCR primers 185 bp
Distal FDR primers 191 bp
Human chromosome 16
16q
16q23
FRA16D
F16D primers
237 bp
Wash
-/+ Aphidicolin (0.25 μm; 48 h)
ChIP using XRCC3, XRCC2, RAD51C and REQ1 antibodies
FRA3B
FRA16D
Mock
Aph
ChIP
FCR
FDR
RAD51C
XRCC2
XRCC3
RECQ1
Input
C
Exponential
HU
FRA3B-FCR
FRA3B-FDR
Fold Enrichment
0 2 4 6 8 10 12 14
0 1 2 3 4 5 6 7 8 9
Goat IgG
RAD51C
XRCC2
XRCC3

Sup Figure 4

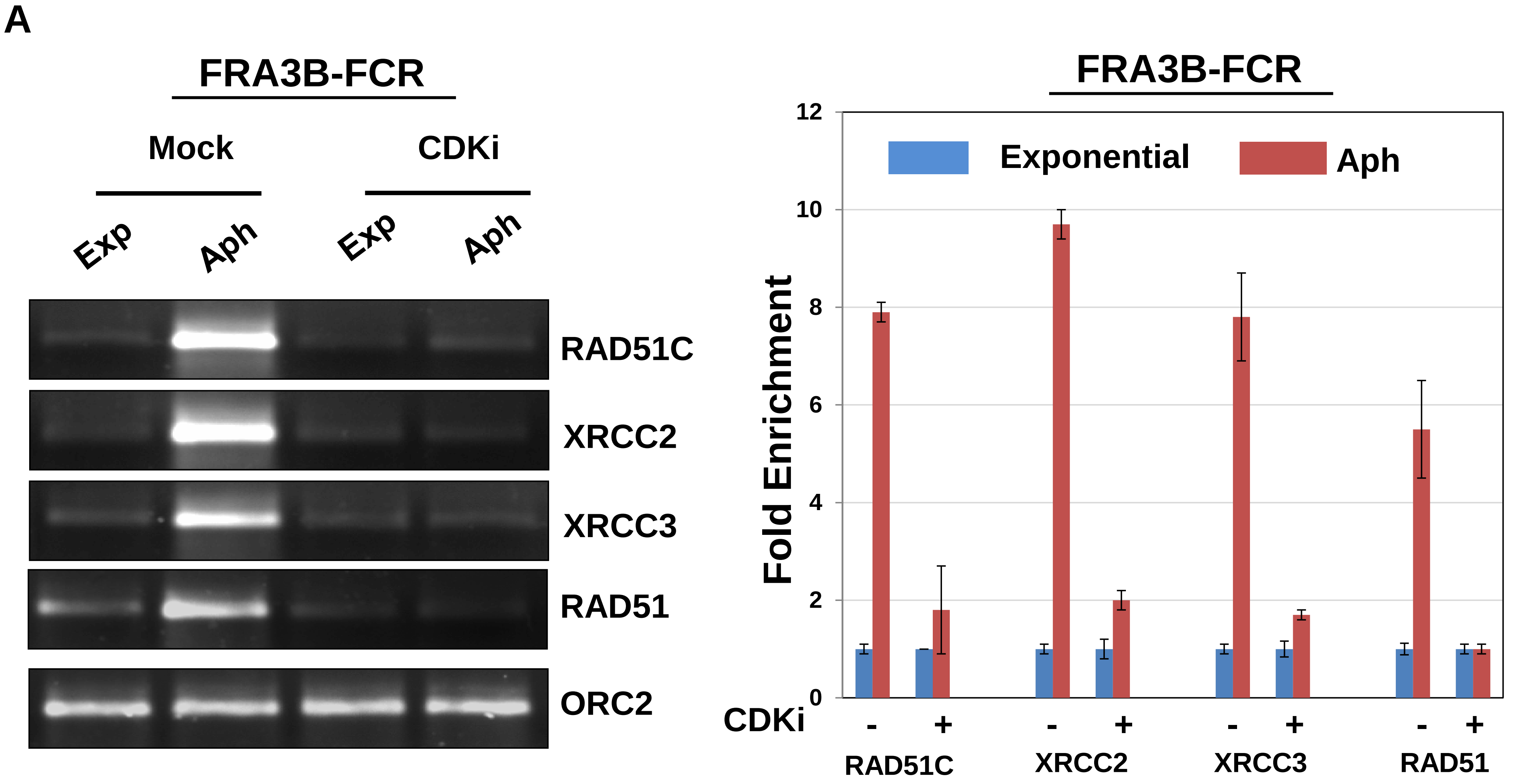
A
FRA3B-FCR
Mock
CDKi
Exp
Aph
Exp
Aph
RAD51C
XRCC2
XRCC3
RAD51
ORC2
FRA3B-FCR
Exponential
Aph
Fold Enrichment
12
10
8
6
4
2
0
CDKi
-
+
-
+
-
+
-
+
RAD51C
XRCC2
XRCC3
RAD51

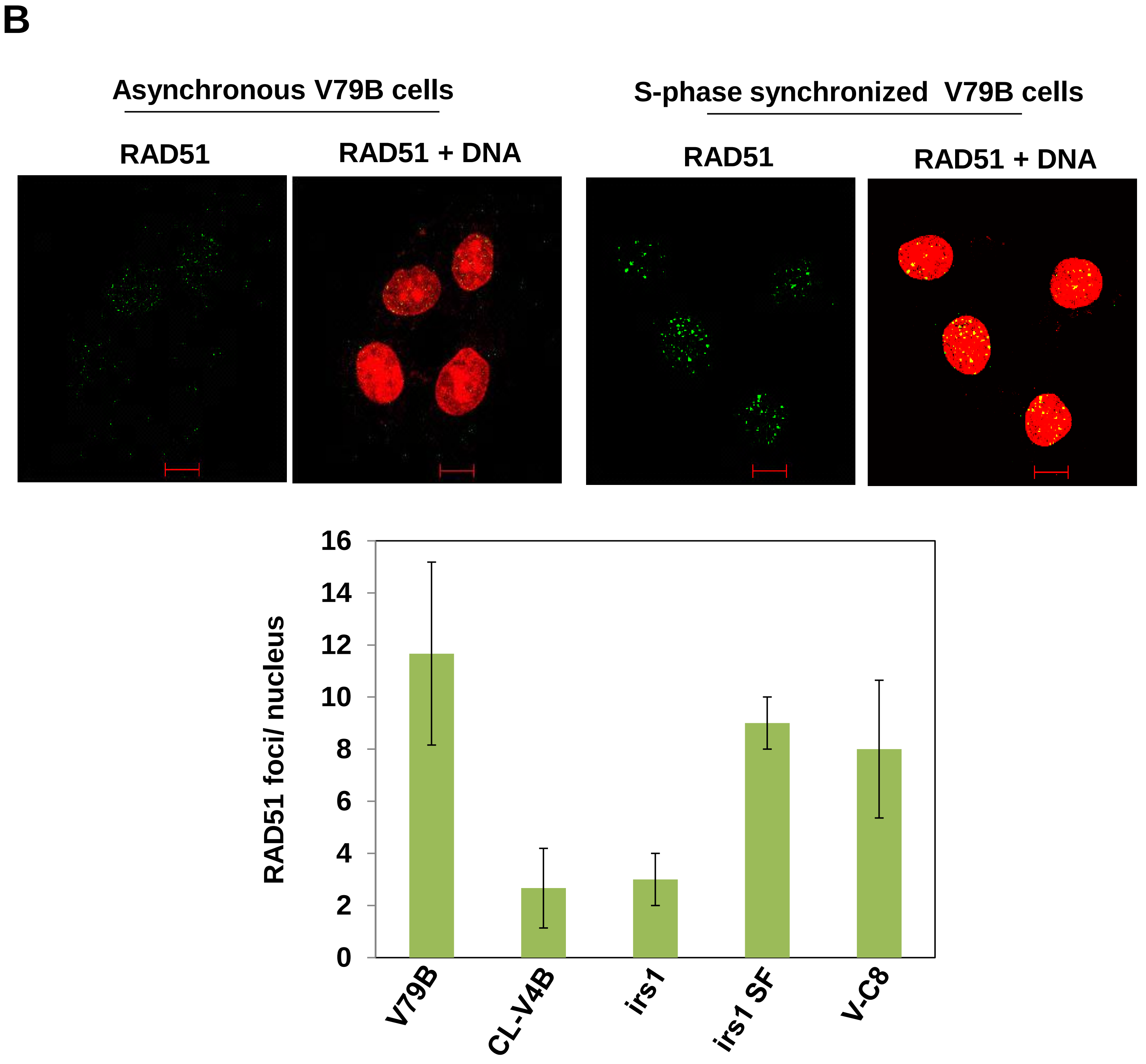
B
Asynchronous V79B cells
S-phase synchronized V79B cells
RAD51
RAD51 + DNA
RAD51
RAD51 + DNA
RAD51 foci/ nucleus
16
14
12
10
8
6
4
2
0
V79B
CL-V4B
irs1
irs1 SF
V-C8

Sup Figure 5

A

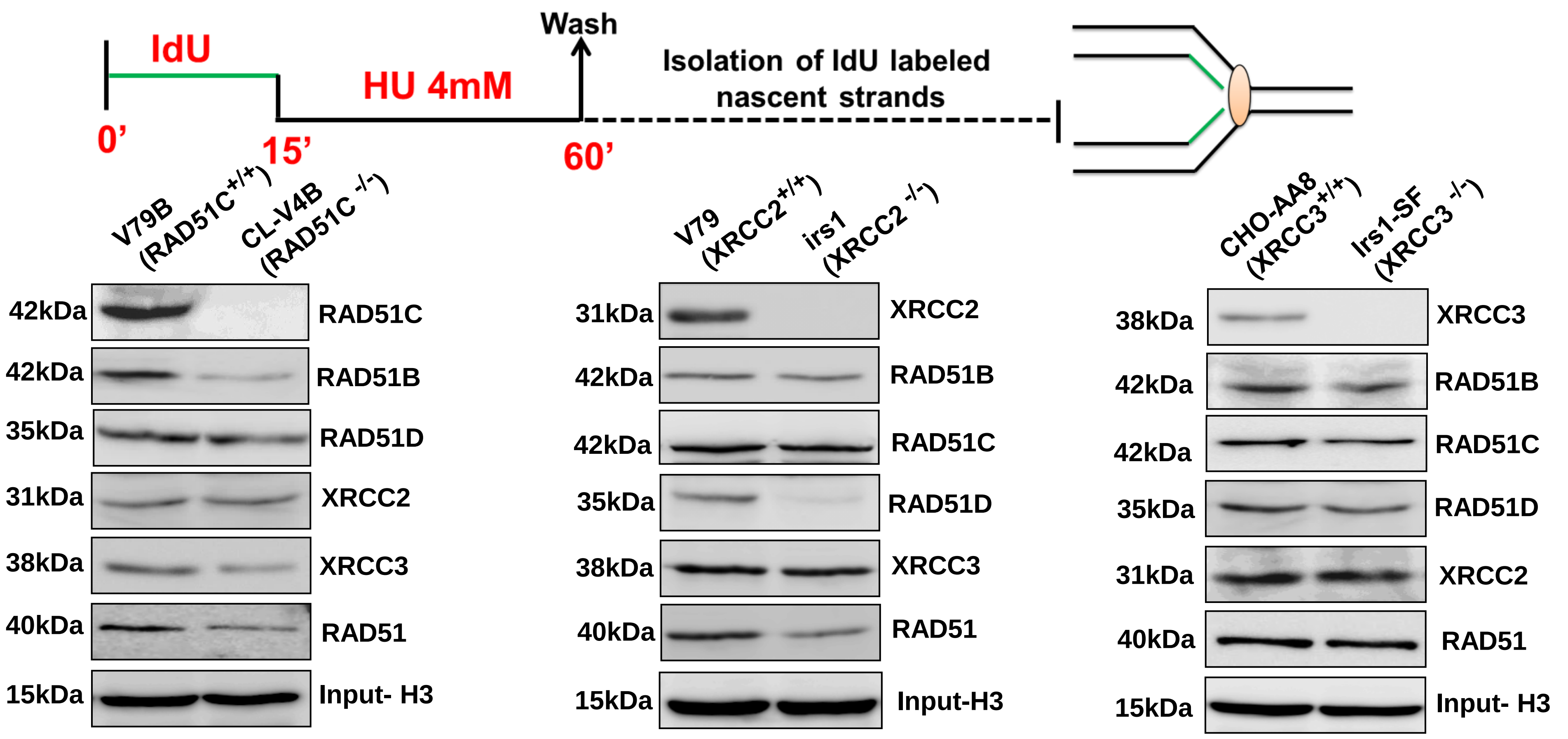

B

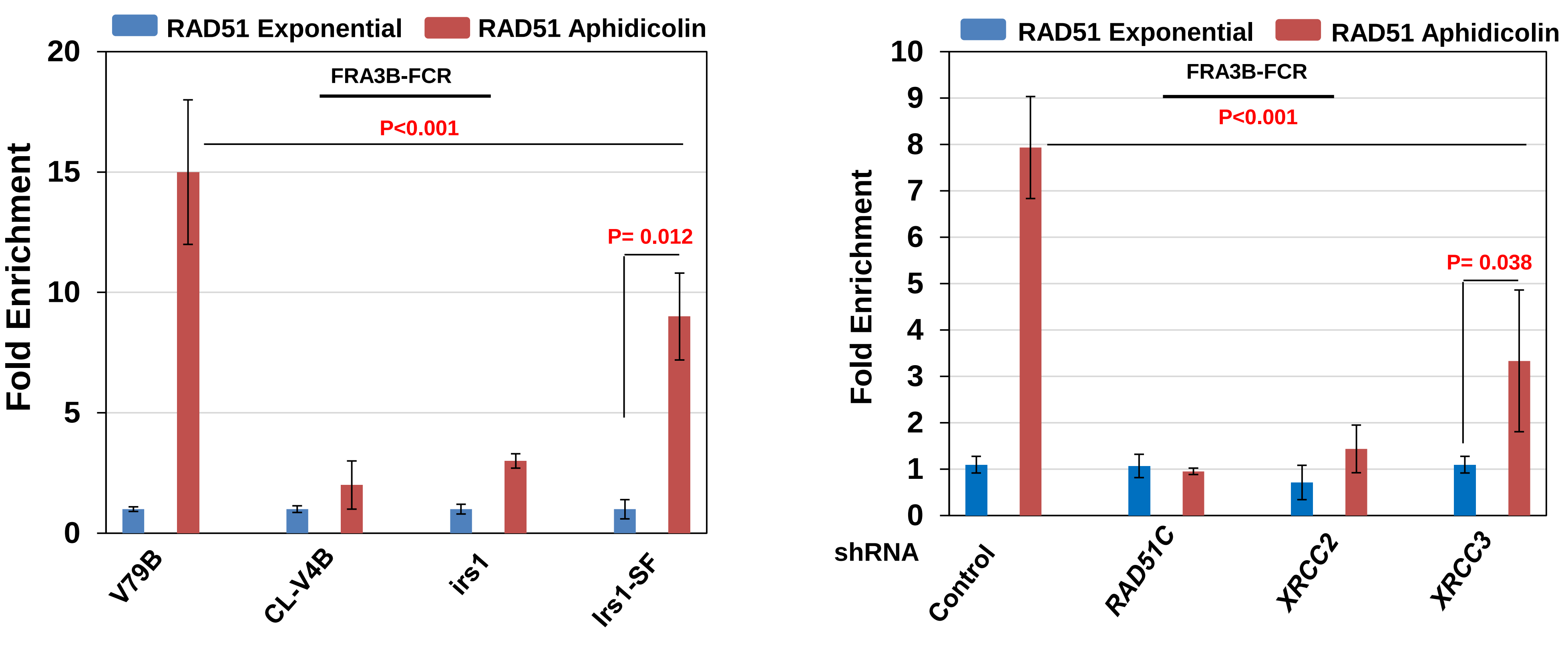

Sup Figure 6

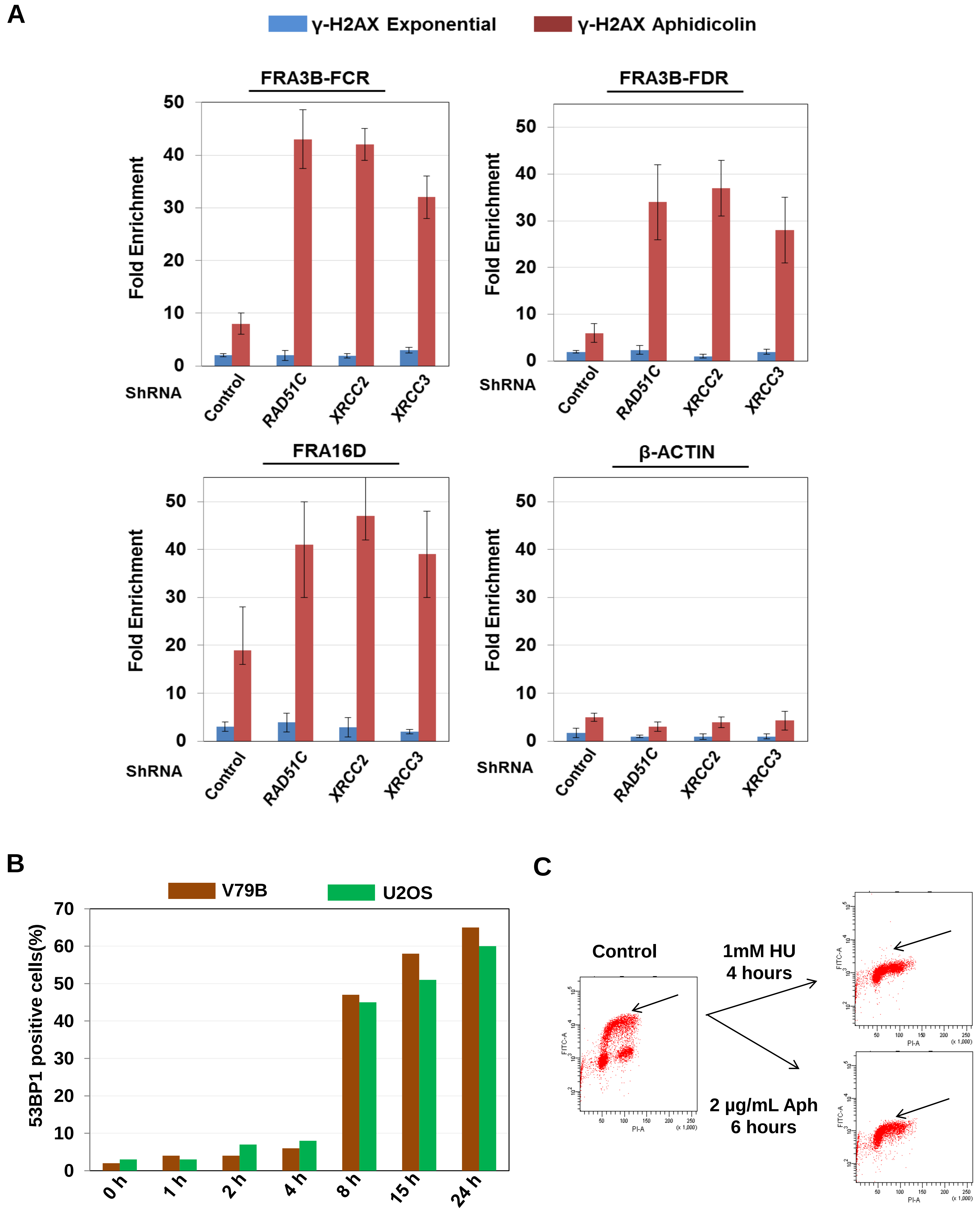
A
γ-H2AX Exponential
γ-H2AX Aphidicolin
FRA3B-FCR
FRA3B-FDR
FRA16D
β-ACTIN
Fold Enrichment
50
40
30
20
10
0
ShRNA
Control
RAD51C
XRCC2
XRCC3
B
V79B
U2OS
53BP1 positive cells(%)
70
60
50
40
30
20
10
0
0 h
1 h
2 h
4 h
8 h
15 h
24 h
C
Control
1mM HU
4 hours
2 μg/mL Aph
6 hours
FITC-A
PI-A

Sup Figure 7

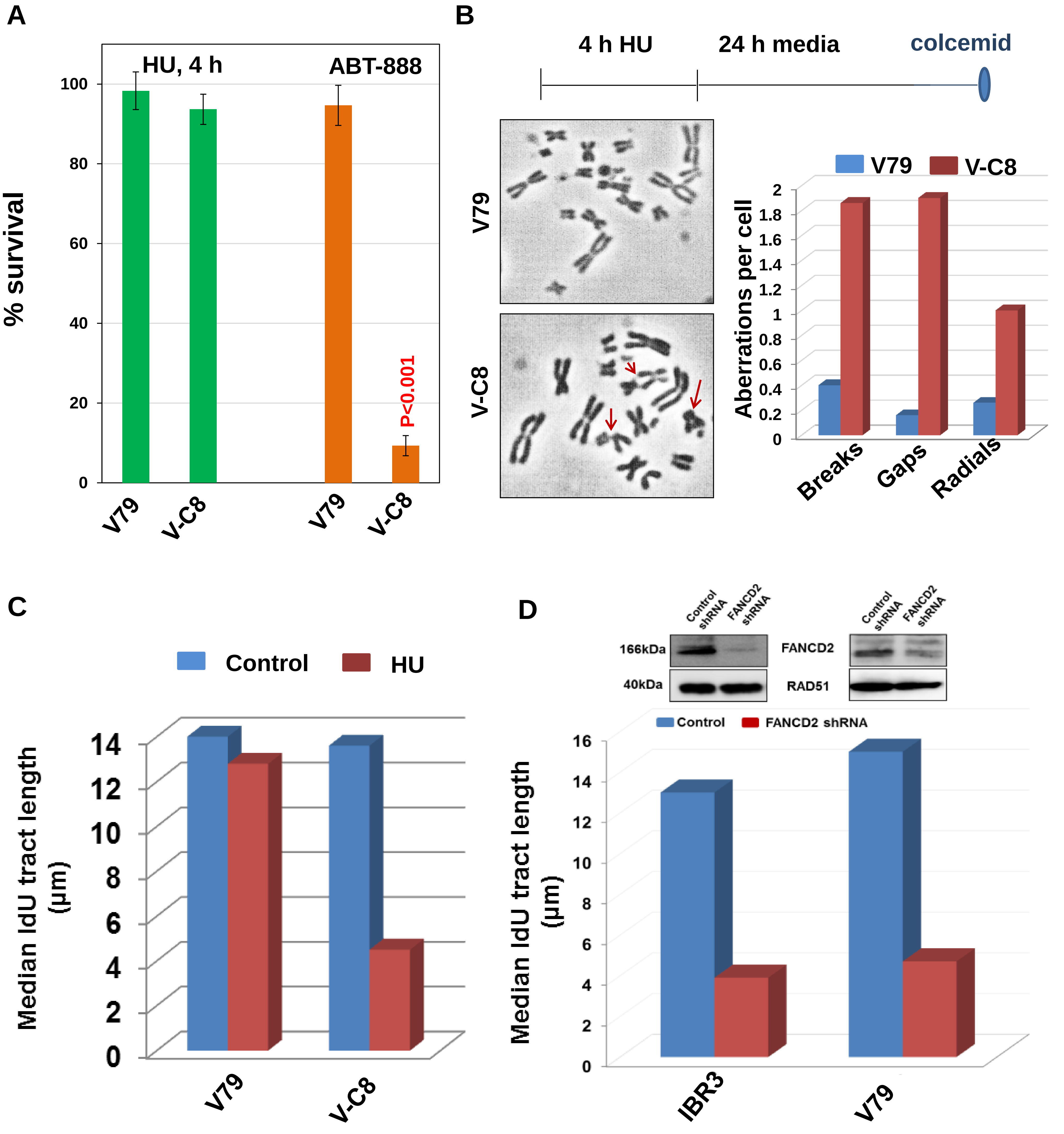

A
HU, 4 h
ABT-888
% survival
P<0.001
V79
V-C8
V79
V-C8
B
4 h HU
24 h media
colcemid
V79
V-C8
V79
V-C8
Aberrations per cell
Breaks
Gaps
Radials
C
Control
HU
Median IdU tract length (μm)
V79
V-C8
D
Control shRNA
FANCD2 shRNA
Control shRNA
FANCD2 shRNA
166kDa
FANCD2
40kDa
RAD51
Control
FANCD2 shRNA
Median IdU tract length (μm)
IBR3
V79

Sup Figure 8

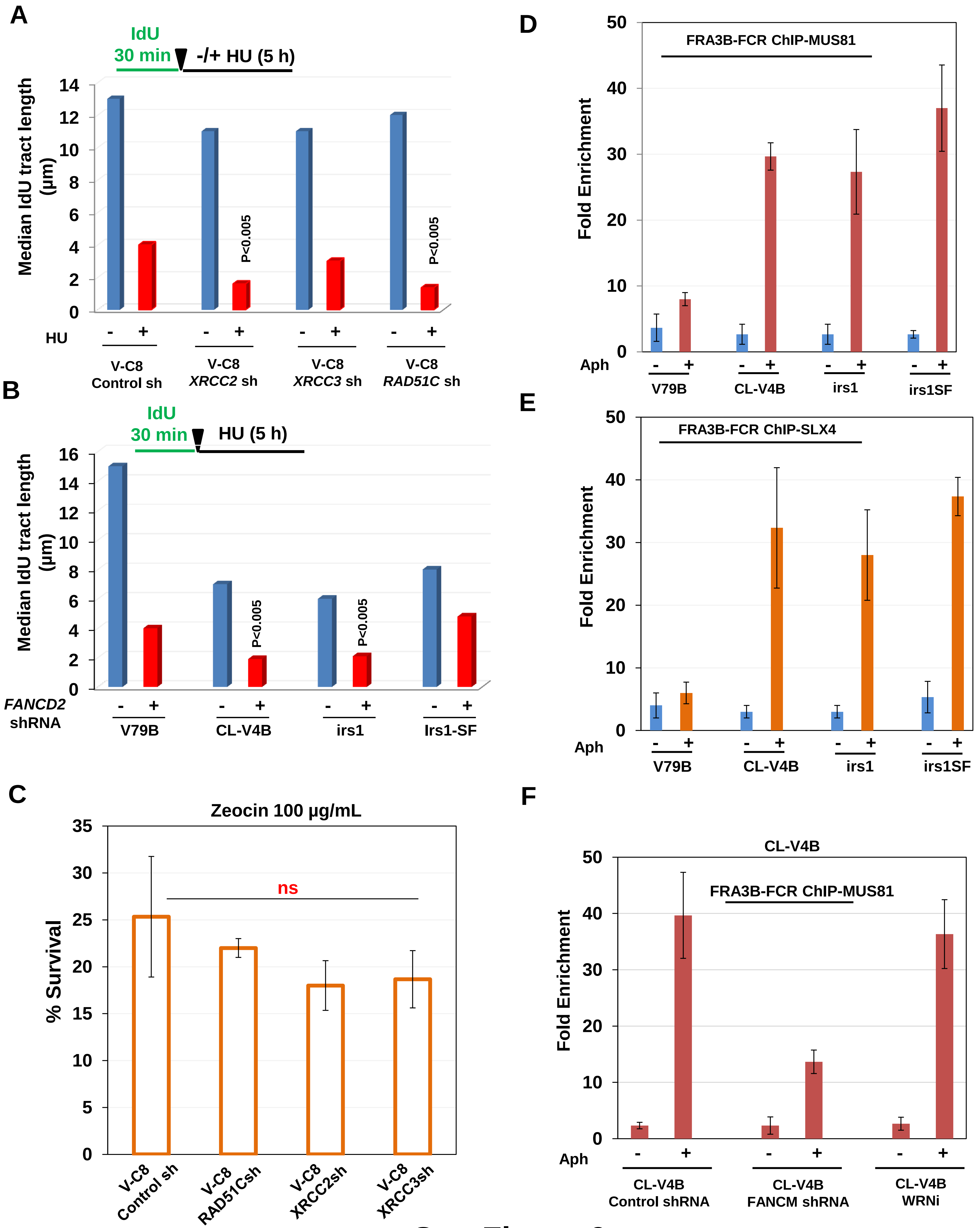

A
IdU
30 min
-/+ HU (5 h)
Median IdU tract length (µm)
14
12
10
8
6
4
2
0
P<0.005
P<0.005
HU
- +
- +
- +
- +
V-C8
Control sh
V-C8
XRCC2 sh
V-C8
XRCC3 sh
V-C8
RAD51C sh
B
IdU
30 min
HU (5 h)
Median IdU tract length (µm)
16
14
12
10
8
6
4
2
0
P<0.005
P<0.005
FANCD2 shRNA
- +
- +
- +
- +
V79B
CL-V4B
irs1
Irs1-SF
C
Zeocin 100 µg/mL
% Survival
35
30
25
20
15
10
5
0
ns
V-C8 Control sh
V-C8 RAD51Csh
V-C8 XRCC2sh
V-C8 XRCC3sh
D
FRA3B-FCR ChIP-MUS81
Fold Enrichment
50
40
30
20
10
0
Aph
- +
- +
- +
- +
V79B
CL-V4B
irs1
irs1SF
E
FRA3B-FCR ChIP-SLX4
Fold Enrichment
50
40
30
20
10
0
Aph
- +
- +
- +
- +
V79B
CL-V4B
irs1
irs1SF
F
CL-V4B
FRA3B-FCR ChIP-MUS81
Fold Enrichment
50
40
30
20
10
0
Aph
- +
- +
- +
CL-V4B
Control shRNA
CL-V4B
FANCM shRNA
CL-V4B
WRNi

Sup Figure 9

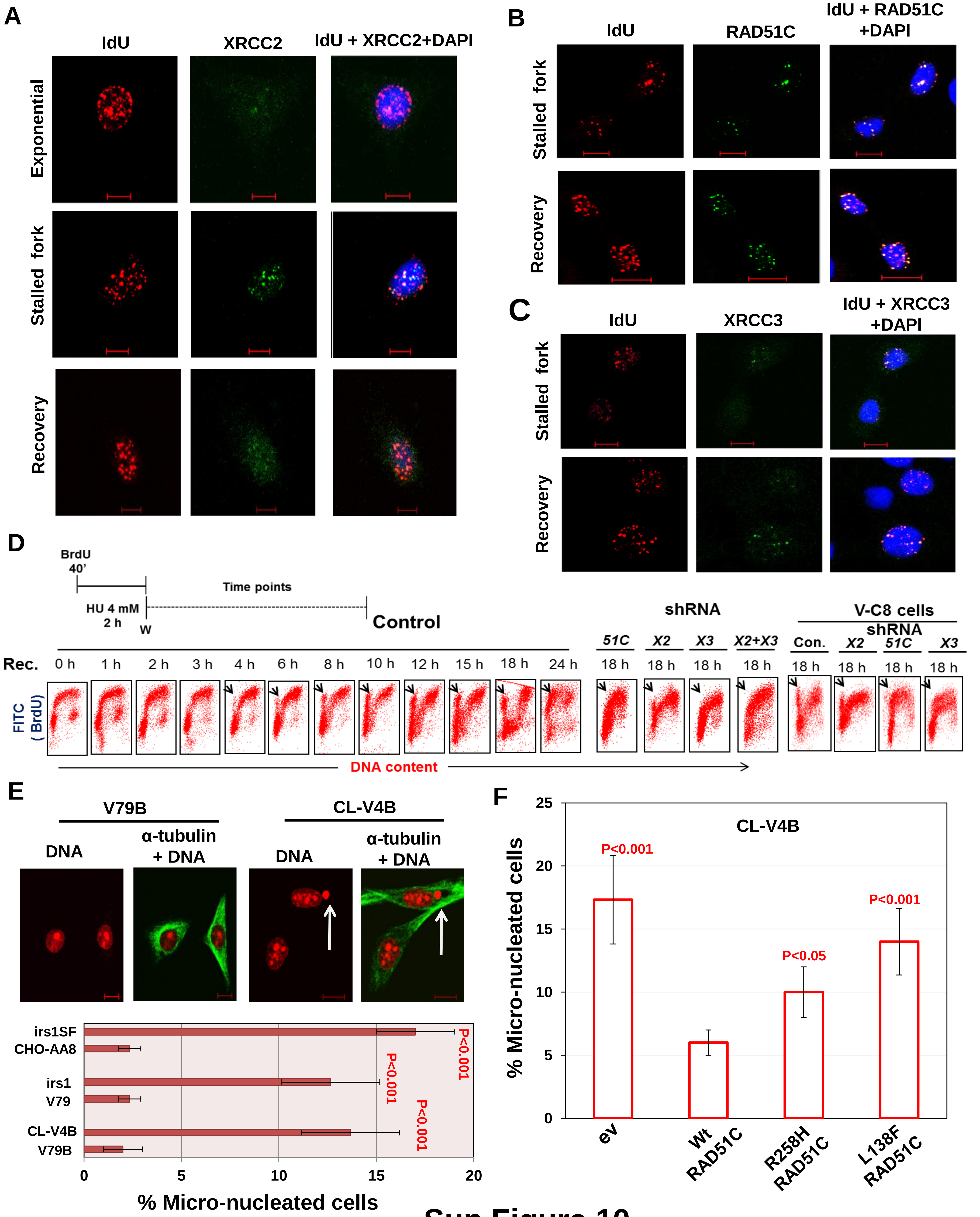

Sup Figure 10
